# Universally Conserved Relationships between Nuclear Shape and Cytoplasmic Mechanical Properties in Human Stem Cells

**DOI:** 10.1038/srep23047

**Published:** 2016-03-15

**Authors:** Oswaldo A. Lozoya, Christopher L. Gilchrist, Farshid Guilak

**Affiliations:** 1 Epigenetics and Stem Cell Biology Laboratory, National Institute of Environmental Health Sciences, Research Triangle Park, NC 27709, USA; 2 Department of Biomedical Engineering, Duke University, Durham, NC 27708, USA; 3 Departments of Orthopaedic Surgery, Biomedical Engineering, and Developmental Biology, Washington University in St. Louis, St. Louis, MO 63110, USA; 4 Shriners Hospitals for Children - St. Louis, St. Louis, MO 63110, USA.

## Abstract

The ability of cells to proliferate, differentiate, transduce extracellular signals and assemble tissues involves structural connections between nucleus and cytoskeleton. Yet, how the mechanics of these connections vary inside stem cells is not fully understood. To address those questions, we combined two-dimensional particle-tracking microrheology and morphological measures using variable reduction techniques to measure whether cytoplasmic mechanics allow for discrimination between different human adherent stem cell types and across different culture conditions. Here we show that nuclear shape is a quantifiable discriminant of mechanical properties in the perinuclear cytoskeleton (pnCSK) of various stem cell types. Also, we find the pnCSK is a region with different mechanical properties than elsewhere in the cytoskeleton, with heterogeneously distributed locations exhibiting subdiffusive features, and which obeys physical relations conserved among various stem cell types. Finally, we offer a prospective basis to discriminate between stem cell types by coupling perinuclear mechanical properties to nuclear shape.

The nucleus and cytoskeleton are physically connected in eukaryotic cells. These connections allow cells to gather physical information about their surroundings using their cytoskeleton, and then relay it to the nucleus where it elicits physiological responses^1^,^2^,^3^. How these connections elicit a response from the nucleus depends on the phenotype of the cell and the cytokines it is exposed to in its local microenvironment^4^,^5^,^6^.

The ability of cells to perceive and respond to physical stimuli exists throughout development. This trait depends not only on the mechanical properties of the different cytoskeletal networks, but also on their ability to be remodeled under stress, as well as the interaction between the cytoskeleton and the nucleus^7^,^8^. Cells harness the mechanical information reaching this anchorage to tune their phenotype during development and to coordinate their nuclear state with their microenvironment^9^,^10^. Through these interactions, cells can organize higher-level morphogenic mechanisms such as collective cell migration^11^,^12^,^13^,^14^,^15^ and differential sorting^16^,^17^,^18^,^19^; over time, these phenomena prescribe morphogenesis, stem cell differentiation and tissue heterogeneity^20^,^21^,^22^,^23^,^24^,^25^.

Stem cells can reorganize their cytoskeleton to regulate intracellular mechanics during differentiation and to adapt to changing physical and biochemical environments. During cytoskeletal remodeling, cells reshuffle their cytoskeletal anchorage to the nucleus to continue sensing their surroundings while remodeling their intracellular architecture; meanwhile, the nucleus may adapt its anatomy to support a changing cytoskeleton^26^. Hence, the nuclear shape represents an architectural fingerprint that evokes a balance between mechanics of the nucleus–which senses mechanical signals from the cell’s microenvironment–and the cytoskeleton–which is responsible for relaying those mechanical signals across the cell–in cells physically coupled to their surroundings. This structural coupling lies beneath the correlation between nuclear shape and multipotency usually observed in stem cells *in vitro*^25^,^26^,^27^,^28^,^29^,^30^.

Structural changes lead to directional and heterogeneous physiology in the nucleus and cytoplasm of stem cells during differentiation^31^,^32^,^33^,^34^; thereafter, those specialized structural properties can guide initially sparse pools of progenitors to assemble physiologically sophisticated tissues, with complex multicellular makeup and spatially regulated architectures^18^,^35^,^36^,^37^,^38^,^39^. Seen under this light, it seems clear that the cytoskeleton may show distinctive structural features among different stem cell types, yet we still ignore how these distinctions are expressed in the intracellular mechanics of stem cells or how can they be leveraged to discriminate stem cell lineages by physical cues. To address such questions, we examined cytoplasmic mechanics in human stem cells. Our hypothesis was that stem cells with different levels of multipotency–or “stemness”–also show discernible cytoplasmic mechanics; in other words, we tested whether the mechanical properties inside the cytoplasm are practical measures that can discriminate between different stem cell phenotypes, in particular by asking if the mechanical properties inside the cytoplasm: a) captured the observed structural reorganization of the cytoskeleton after exposing stem cells to soluble cytokines or disrupting their actin polymerization pharmacologically *in vitro*; b) adapted differently to induced cytoskeletal remodeling when stem cell morphology was prescribed into a fixed geometry; and c) could distinguish among stem cells from different lineages. After determining associations between cytoplasmic mechanics in stem cells and their cytoskeleton, we examined if *a combination* of those mechanical properties followed a predictive relation common to stem cells from all experimental regimes–with the presumption that, if extant, such relations may hint at a structural foundation present in all stem cells.

## Results

### Localization of intracellular beads within F-actin networks in adherent stem cells

**Figure 1:**
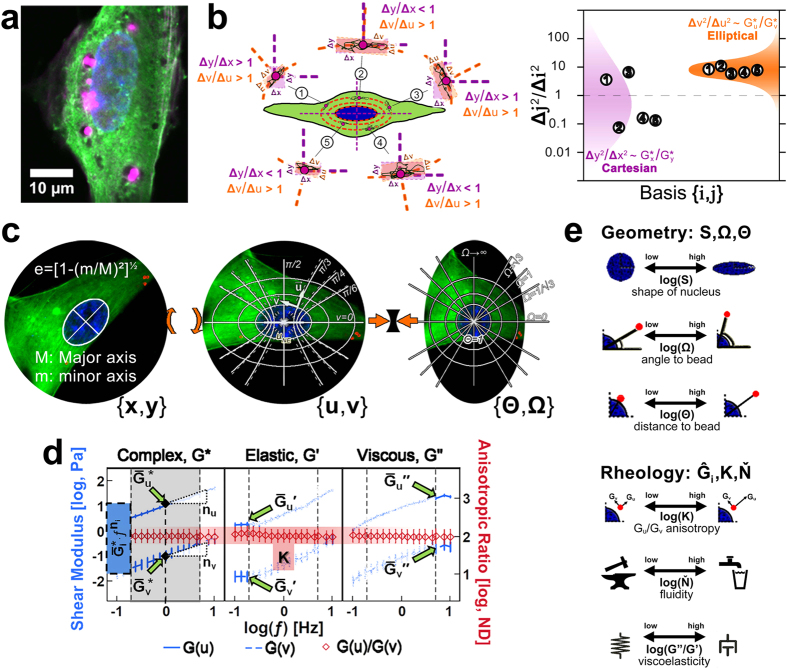
A nucleus-centered elliptical coordinate system for perinuclear cytoskeleton (pnCSK) rheology. (**a**) Four-channel laser confocal microscopy of paraformaldehyde-fixed hASCs on fibronectin-coated coverslip cultures expressing an eGFP-actin fusion protein (green). Cells contain intracellular AlexaFluor 568-tagged beads (1-μm diameter, red) delivered by lipid-based endocytosis in culture. After fixation, cells were stained with Hoechst 33342 (blue) and phalloidin-AlexaFluor 633 (magenta) to highlight localization of nucleus and F-actin fibers, respectively. (**b**) Representative cell diagram depicting differences observed in dispersion of anisotropic mean squared displacements ≪Δr^2^(τ)≫ measured using either rectangular coordinates {*x*, *y*} or curvilinear nucleus-centered elliptical coordinates {*u*, *v*}. (**c**) Nondimensional parameterization of intracellular bead position in nucleus-centered elliptical coordinates. Bead coordinates are circularized relative to the nucleus of each cell by normalization to the locus *u*_*NE*_ of its nuclear perimeter in elliptical coordinates; after normalization, nucleus-relative bead distances and angular pitch {**Θ**, **Ω**} are ensembled into a unified polar nondimensional map in which all nuclear perimeters trace along **Θ** = 1. (**d**) Parameterization of anisotropic intracellular rheology via a model power-law rheology model 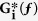 = 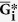
**ƒ**^**n**^_**i**_ for **i** = {*u*, *v*}. Complex shear moduli 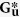 and 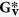 are decomposed into their elastic and viscous terms **G′** and **G″**. In the frequency range 0.2 Hz < **ƒ**_P_ < 5 Hz (i.e. −0.7 < log (**ƒP**) < 0.7, where **ƒP** is the power-law frequency span) anisotropic rheology exhibits constant flow behavior indices **n**_**u**_ **=** **n**_**v**_, constant damping ratio **G″/G′** = tan[α∙π/2], and constant ratio between flow consistency indices **K** = 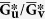; outside **ƒP** , intracellular rheology displayed constant **G**_**i**_**′** for **ƒ** < 0.2 Hz and constant **G**_**i**_**″** for **ƒ** > 5 Hz. Altogether, 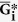, **n**_**i**_ and **G″/G′** within **ƒP** for **i** **=** **{***u*, *v*} in conjunction with moduli **G**_**i**_**′(ƒ** < 0.2 Hz) and **G**_**i**_**″**(**ƒ** > 5 Hz) suffice to recapitulate anisotropic cytoplasmic rheology in curvilinear elliptical coordinates. (**e**) Geometric (**S**, **Ω**, **Θ**) and rheological (**K**, **Ň**, **G″/G′**) nondimensional parameters derived from **e**lliptical coordinate characterization of intracellular mechanics shown in (**c,d**).

We characterized cytoplasmic mechanics in live human stem cells by particle-tracking microrheology (PTM). We used 1-μm spherical beads delivered by endocytosis as tracking probes. This approach has been justified by other groups before, showing that estimates of cytoplasmic mechanics in live cells are comparable using beads 1 μm or larger for microrheology, whether enclosed inside or outside endosomes^40^. In our experiments, we opted for an optimized low-titer bead lipofection strategy that minimized detrimental effects on cell viability and growth in our cultures (see Methods and Supplementary Discussion for details). After introducing tracking beads in human stem cells, we performed confocal microscopy to assess whether cytoplasmic beads were entangled inside cytoskeletal lattices or segregated within cytoplasmic vacuoles. Both live microscopy with actin-GFP expressing cells and fixed-cell microscopy with phalloidin staining revealed subsets of single beads with dense F-actin colocalization along their periphery but not inside vacuoles (Fig. 1a). These observations suggested that, after endocytosis, some beads may still be useful to approximate cytoskeletal microrheology in live stem cells when entangled within F-actin lattices.

### Parameterized intracellular rheology in a nucleus-centered elliptical coordinate system

Displacements of nonvacuolated cytoplasmic beads showed a clear directional bias. This behavior was expected, since the cytoskeleton is a structural network of highly oriented filaments that exhibits anisotropic mechanics. Interestingly, we also identified that the direction of largest bead displacements was roughly parallel to the nuclear surface and consistent in all cells tested (see illustration in Fig. 1b). These results suggested that anisotropic bead motion obeyed an orthotropic basis, particularly one based on the nuclear geometry. This interpretation harbors important statistical outcomes for bead motion analysis: if adequate, an orthotropic model can highlight anisotropic differences in averaged space-dependent properties that a fixed rectangular coordinate system would smear out instead; indeed, we confirmed that a nucleus-centered geometric transformation captured the anisotropic behavior of bead displacements better than rectangular coordinates (as illustrated in Fig. 1b). Thus, we adopted a curvilinear and nucleus-centered elliptical coordinate system to analyze individual bead displacements and estimate direction-dependent cytoplasmic properties (Fig. 1c).

The generalized Stokes-Einstein relation (GSER) describes the relation between thermally driven motion of small particles inside a complex fluid and the resisting drag by the surrounding fluid to the particle’s motion. This theory has been widely adopted to approximate the mechanical properties inside live cells by tracking displacements of individual embedded particles^31^,^40^,^41^,^42^,^43^,^44^,^45^,^46^,^47^,^48^,^49^,^50^,^51^. We used this technique to resolve anisotropic effects in the cytoplasmic mechanics of live adherent stem cells. First, we decomposed the displacements of each bead into their two orthotropic components from nuclear-centered elliptical coordinates; next, we identified those beads whose mean-squared displacement profiles resembled subdiffusive motion in both orthotropic directions; and finally, we estimated subdiffusive rheological properties matching the orthotropic displacements of each bead at its specific location within the cytoplasm. The mathematical subdiffusive criterion we used to select beads for rheological characterization was based on the mean squared displacement power coefficient α(τ) ≡ ∂[log (≪Δr^2^(τ)≫)]/∂[log (τ)], i.e. the slope α(τ) for the mean squared displacement ≪Δr^2^(τ)≫ with respect to tracking time intervals τ in logarithmic space. We collected displacement profiles of beads with α(τ) ≤1 for all τ, which is reminiscent of subdiffusive motion, and traced them back to their cytoplasmic localization in cells; for brevity, we coined the term subdiffusive loci (sDL) in reference to those locations, distinguishing them from those with α(τ) >1 for which GSER is mathematically invalid.

While we recognize that the cytoskeleton is highly dynamic at the molecular level, we also appreciate that it must fulfill a necessarily steady structural role at the cellular scale to enhance tissue assembly; this is a particularly relevant task the cytoskeleton fulfills during early development, when extracellular matrix is scarce and cellular polarization is a critical cue from within cells to guide tissue morphogenesis. The reasoning behind extending GSER theory to describe intracellular mechanics in live stem cells does not speak to any expectations for thermally-driven cytoplasmic properties–in fact, the overall non-Brownian nature of cytoskeletal mechanics is very well documented^41^,^42^,^43^–instead, it aims at capturing short-lived windows of individual bead motion with subdiffusive features, at specific locations within the cytoplasm, and particular to the instant when they were measured. By the same token, this notion also implies that the type of motion exhibited by each bead in particular as it is measured can differ between measurements, testing intervals, and over time. Our experience was consistent with such expectations: beads exhibiting subdiffusive motion at a given 30-s span did not necessarily display a subdiffusive profile in later timepoints; for that reason, we recorded all beads under view and tracked each of them individually and in every testing interval, established whether their motion profiles were consistent with subdiffusive motion during data processing in each case, and managed our data set based on *instances* of local subdiffusive features instead of beads *per se*. We posit that the underlying mechanical properties of cytoskeletal networks can be approximated in this manner. When carried out in many live cells, this strategy also offers other advantages: for example, it yields estimates for observed rheological properties inside the cytoplasm of single live cells that can be tracked in space and over time at physiological scales. Also, we gather enough instances of subdiffusive motion to generate statistical distributions of mechanical properties and use them to discern between cell populations; this is more efficient than looking for cytoplasmic beads that exhibit permanent subdiffusive properties–which is unlikely to occur inside living cells.

**Figure 2:**
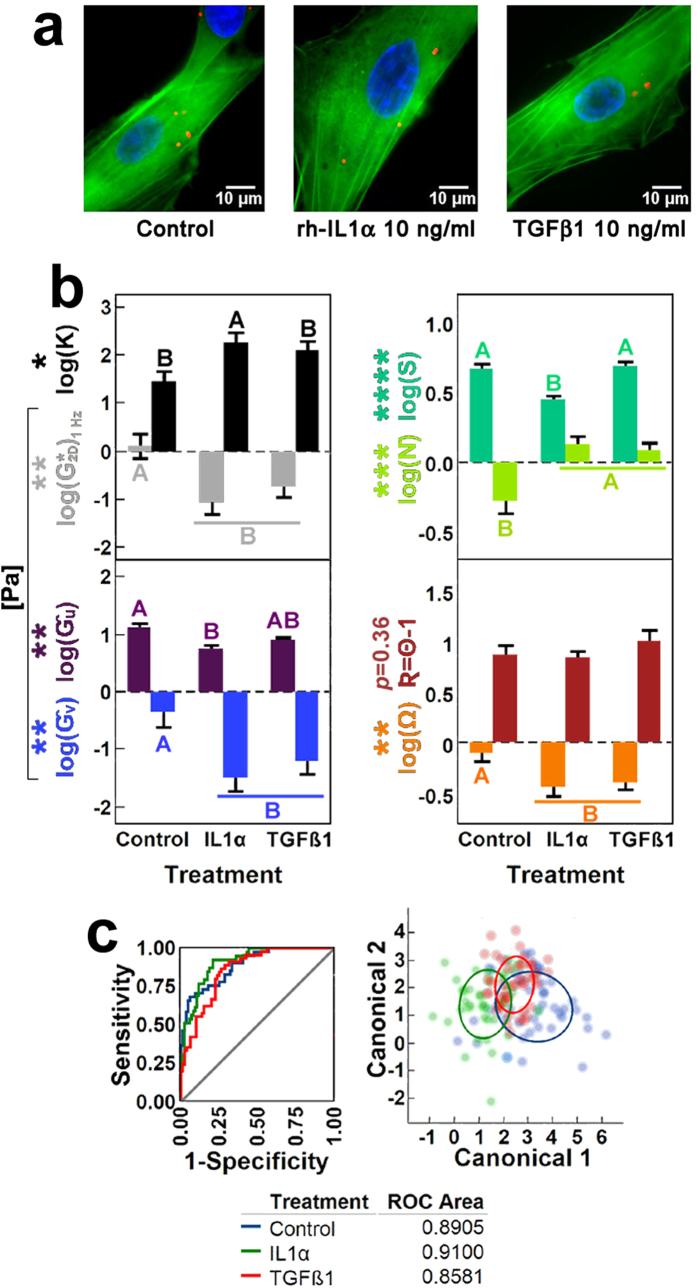
Rheological effects of catabolic and anabolic induction on hASCs with unconstrained morphology at subdiffusive loci (sDL) of the pnCSK. (**a**) Examples of unconstrained hASC morphologies after 1-hr stimulation with catabolic [10 ng/ml rh-IL1α] and anabolic [10 ng/ml hPD-TGFβ1] cytokines. (**b**) Significant changes on geometric and rheological parameters describing altered hASC pnCSK mechanics after treatment with either rh-IL1α or hPD-TGFβ1. (**c**) Discriminant analysis of cytokine-induced cytoskeletal mechanics in hASCs with unconstrained shape in control media and following supplementation with rh-IL1α or hPD-TGFβ1. Error bars depict mean ± s.e.m interval (parameter effect significance: *p < 0.05, **p < 0.01, ***p < 0.001, ****p < 0.0001). Groups not sharing letters within a parameter are statistically significant (paired Tukey-Kramer *post hoc*: *α* < 0.05, >90% power). Receiver operating characteristic (ROC) curves and 50% density ellipses are specific to each independent group within experiments.

**Figure 3:**
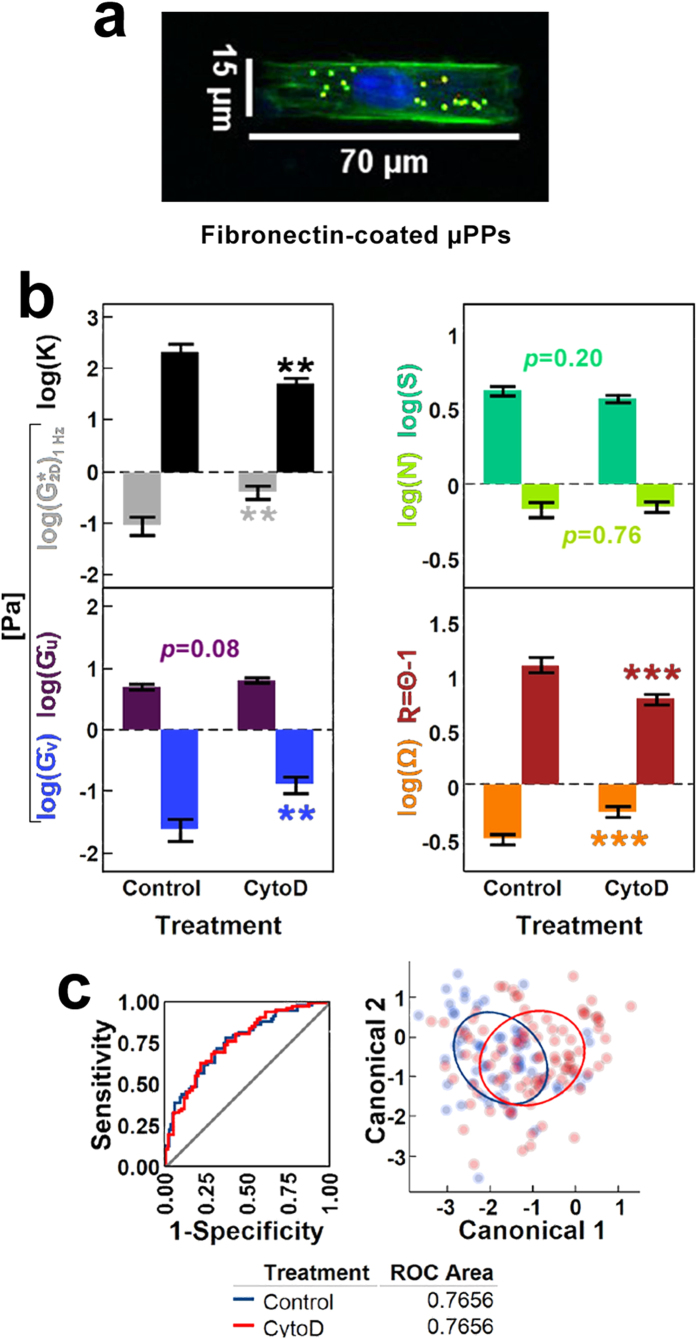
Rheological effects of blocked F-actin polymerization on hASCs with micropatterned (μPP) morphology at subdiffusive loci (sDL) of the pnCSK. (**a**) Morphological constraint of hASCs via fibronectin-coated microablated PVA-film patterning (15 × 70 μPPs). (**b**) Significant changes on geometric and rheological parameters describing altered pnCSK mechanics in hASCs on μPP (15 μm × 70 μm rectangle) with or without 0.5 μM Cytochalasin D (CytoD) supplementation. (**c**) Discriminant analysis of control v. CytoD-induced cytoskeletal mechanics in hASCs with μPP-directed morphology. Error bars depict mean ± s.e.m interval (parameter effect significance: *p < 0.05, **p < 0.01, ***p < 0.001, ****p < 0.0001). Groups not sharing letters within a parameter are statistically significant (paired Tukey-Kramer *post hoc*: *α* < 0.05, >90% power). Receiver operating characteristic (ROC) curves and 50% density ellipses are specific to each independent group within experiments.

**Figure 4:**
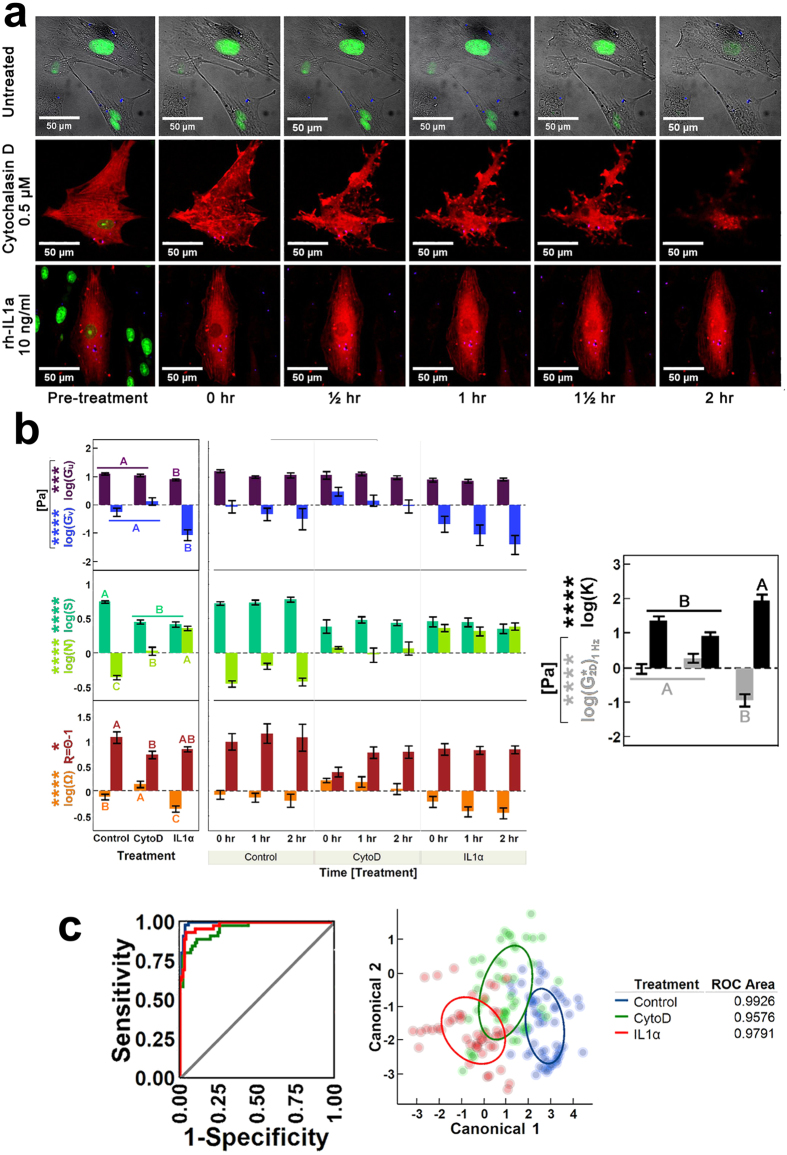
Remodeling of pnCSK under steady-state inflammatory signaling. (**a**) Time course of induced cytoskeletal remodeling by continuous supplementation with either 10 ng/ml rh-IL1α or 0.5 μM CytoD in unconstrained hASCs. (**b**) Significant effects over time from each treatment on geometric and rheological parameters of hASC pnCSK mechanics. (**c**) Discriminant analysis of treatment-induced pnCSK mechanics in hASCs with unconstrained morphology after supplementation with either 10 ng/ml rh-IL1α, 0.5 μM CytoD or none. Parameter significance was confirmed via Time[Treatment] nested two-way ANOVA on log-transformed data (Time *p* > 0.05); error bars depict the mean ± s.e.m interval (parameter effect significance with respect to treatment: *p < 0.05, **p < 0.01, ***p < 0.001, ****p < 0.0001). Groups not sharing letters within a parameter are statistically significant (paired Tukey-Kramer *post hoc*: *α* < 0.05, >90% power). Receiver operating characteristic (ROC) curves and 50% density ellipses are specific to each independent group.

**Figure 5:**
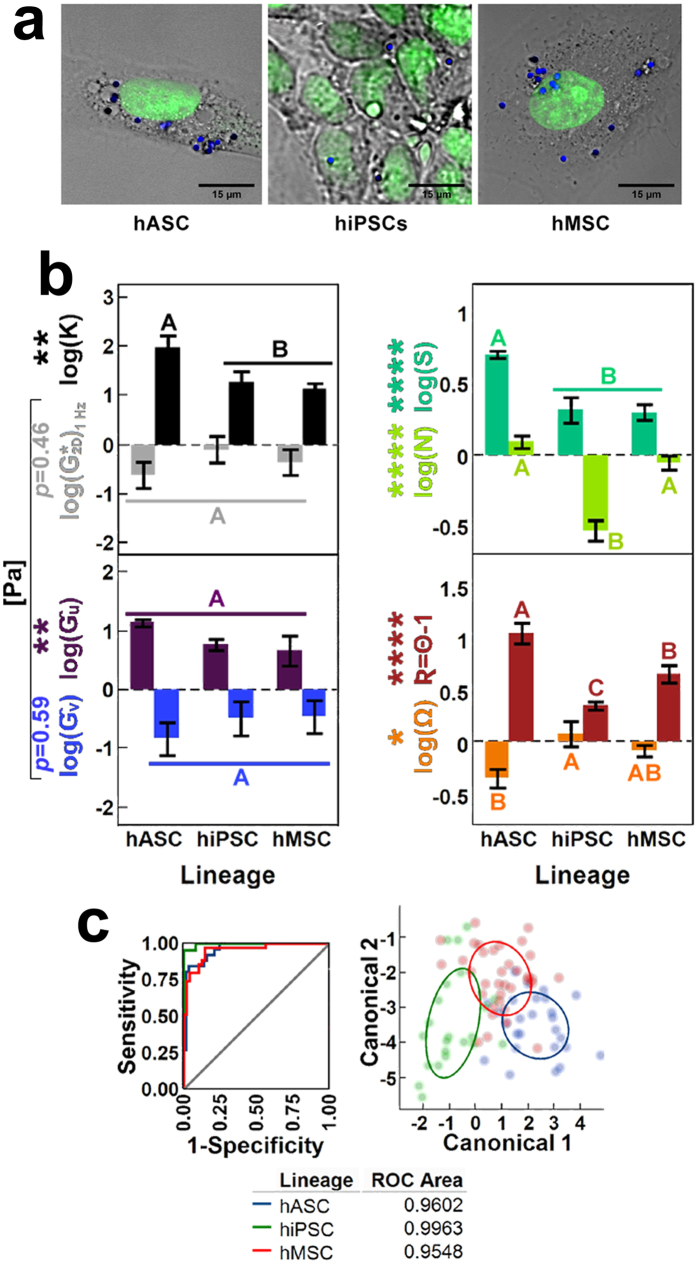
Mechanical signatures in multipotent human cells. (**a**) Representative microscopy of three distinct multipotent cell types (hASCs, hMSCs and hiPSCs) mechanically characterized by pnCSK microrheology (blue: 1-μm diameter AlexaFluor 633-tagged beads, green: SYTO13 nuclear dye). (**b**) Differential pnCSK mechanics among multipotent cell types shown by geometric and rheological parameters. (**c**) Discriminant analysis of multipotent cell types via pnCSK mechanical characterization. Error bars depict the mean ± s.e.m interval (parameter effect significance: *p < 0.05, **p < 0.01, ***p < 0.001, ****p < 0.0001). Groups not sharing letters within a parameter are statistically significant (paired Tukey-Kramer *post hoc*: *α* < 0.05, >90% power). Receiver operating characteristic (ROC) curves and 50% density ellipses are specific to each independent group.

**Figure 6:**
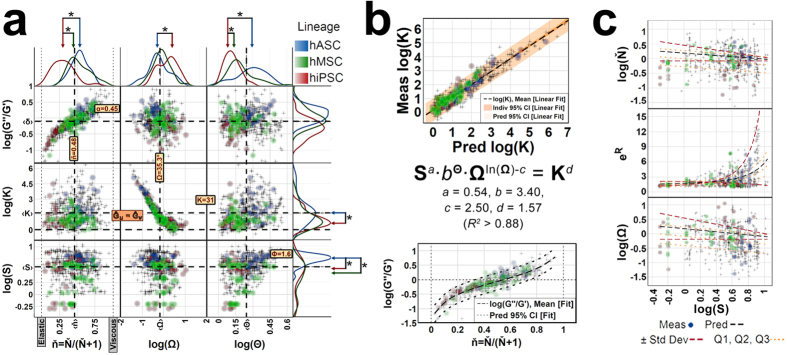
Stem cell pnCSK mechanics are reflected by the nuclear shape. (**a**) Ensemble maps of nondimensional pnCSK mechanics display common multivariate relations independent of exogenous treatments or stem cell types. Samples from experiments comparing different stem cell types (Experiment IV, see Table S1) are highlighted; global medians for parameters **Ω**, **G″**/**G′**, **S**, **K** and **Ň** are shown as calculated from data across all experiments (Experiments I–IV, see Table S1). (**b**) Stem cell type-independent mechanics exhibit an empirical conservative behavior via a latent mixture model of structural pnCSK properties predicted by **S**^*a*^∙*b*^**Θ**^∙**Ω**^ln(**Ω**)−*c*^ = **K**^*d*^ (mean ± 95% confidence interval of prediction, *R*^2^ > 0.89). (**c**) In human stem cells, probabilistic distributions of pnCSK mechanical properties are numerically tractable from the nuclear shape, as predicted by cumulative frequency analyses (see Table 1).

Altogether, joint implementation of GSER theory with nucleus-centered elliptical coordinates revealed that: a) sDL localize predominantly in a region within one nuclear radius from the nuclear perimeter that we refer to as the perinuclear cytoskeleton (pnCSK) (see Figs 2, 3, 4, 5, 6); b) the pnCSK in human stem cells exhibits a constant anisotropic ratio between radial and angular shear moduli; and c) cytoplasmic rheology in live stem cells follows a power-law behavior with frequency-invariant viscoelastic damping in the range 0.2 Hz < **ƒ**_P_ < 5 Hz (Fig. 1d). Like the nucleus-centered elliptical coordinate system (Fig. 1c), power-law rheology is amenable to nondimensionalization; therefore, we reduced the number of variables in our analysis by projecting the rheology and location of every sDL captured in stem cells onto a unifying nondimensional canvas with the following parameters: a) nuclear elliptical shape **S**; b) angle to bead from the major elliptical axis **Ω**; c) distance to bead from the nuclear centroid **Θ**; d) anisotropy **K**; and e) fluidity **Ň**. In addition, we also quantified the damping ratio **G″/G′** at **ƒP** = 1 Hz as a reference measure of viscoelasticity (Fig. 1e; refer to Supplementary Information for further details on our nondimensional analysis).

### Effects of cytokine signaling on hASC cytoskeletal mechanics (Experiment I)

Soluble cytokines are active modulators of cytoskeletal remodeling in adult stem cells such as human adipose-derived mesenchymal stem cells (hASCs). We tested the effects by two different cytokines on hASC cytoplasmic rheology after 1 hr of static culture: IL1α, a catabolic inflammatory cytokine that activates transport of both *de novo* and recycled G-actin monomers from depolymerized F-actin fibers to the cell cortex^4^; and TGFβ1, an anabolic growth factor that promotes cell spreading, F-actin assembly and stress fiber stabilization^52^ (Fig. 2a). Each of these treatments induced significant effects on hASC cytoplasmic rheology compared to untreated conditions, and in all cases such effects were accompanied by reductions in the angular pitch **Ω** of sDL with respect to the major axis of the nucleus, albeit without changing the relative distance **R** = **Θ** − 1 of beads from the nuclear perimeter (where **Θ** = 1 by definition) (Fig. 2b).

Treatment with IL1α led to significant losses in all **Ĝ**_**u**_, **Ĝ**_**v**_ and 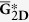 shear moduli and nuclear shape **S** as well as increased fluidity **Ň** and anisotropy **K** (Fig. 2b). Those losses in shear moduli after IL1α treatment were also smaller, although not statistically different, with respect to those induced by TGFβ1 supplementation. Cytoskeletal remodeling after TGFβ1 induction led to structural properties similar to those observed in IL1α-treated hASCs with two exceptions: TGFβ1 treatment had no effect on either nuclear shape **S** or anisotropy **K**, unlike the effects observed after IL1α supplementation which were statistically significant.

Having observed differences in the mechanical profiles induced with each cytokine, we then asked whether the collective of nondimensional pnCSK parameters for each condition contributed to characteristic mechanical signatures. Indeed, multivariate profiling of pnCSK mechanics by discriminant analysis (Fig. 2c) showed good segregation among control hASCs or treated with IL1α and TGFβ1; this is shown by the area under the receiver operating characteristic (ROC) curve from each treatment–a measure of how often guessing the statistical group of a randomly selected cell is expected to match, based exclusively on its observed nondimensional pnCSK mechanics–which surpassed 85% in each condition.

### Cytoskeletal remodeling in hASCs with directed cell morphology (Experiment II)

To quantify relationships between cell morphology and cytoskeletal mechanics, PVA-film micro-photopatterning (μPP) techniques^53^ were used to control *in vitro* morphology of hASCs. Using this technique, we obtained attachment of single hASCs in an array of 15-μm × 70-μm rectangular fibronectin-coated geometries (Fig. 3a). Then, we introduced short-term supplementation with or without cytochalasin D (CytoD), a cytoskeletal disruptor known for its capacity to block F-actin polymerization sites and compromise the structural integrity of cytoplasmic microfilaments^54^,^55^.

Treatment with CytoD impacted pnCSK mechanics significantly as demonstrated by loss of anisotropy **K** driven by a rise in **Ĝ**_**v**_ (Fig. 3b). Along with this mechanical outcome, sDL were found at higher angular pitch **Ω** with lower **R** values, which correspond to a shift in sDL positions that cluster near the nucleus. Notably, those effects on morphologically constrained hASCs were accompanied by no change in nuclear shape **S**, fluidity **Ň**, or radial stiffness **Ĝ**_**u**_. Altogether, CytoD-treated hASCs with patterned morphologies were segregated poorly from untreated ones on the basis of multivariate pnCSK mechanics, as reflected by discriminant analysis yielding areas under the ROC curve less than 76% (Fig. 3c).

### Evolution of pnCSK mechanics during induced remodeling of F-actin in hASCs (Experiment III)

To mimic the time-dependent behavior of pnCSK mechanics in cells under “steady” catabolic induction, we tracked cytoplasmic beads in single cells for 30 seconds at regular 15-minute intervals for 2 hours in hASCs supplemented with either rh-IL1α or CytoD. The concentration in culture was maintained for each treatment by continuously refreshing supplementation media with an open-loop exchange system for *in vitro* cultures. Our experiments showed that both conditions elicited significant cytoskeletal changes in hASCs with active cytoplasmic remodeling for up to 2 hours (Fig. 4a); yet, although significantly distinct across treatments, the effects on fluidity **Ň**, radial modulus **Ĝ**_**u**_, and all morphological parameters (**R**, **S**, and **Ω**) stabilized quickly, at some point between the exchange of growth media with treatment media and the end of the first round of single-cell recordings (about 5–10 min), and persisted thereafter in all cases (treatment *p* < 0.0001, time *p* > 0.93, treatment × time *p* < 0.03, Identity MANOVA). Within this timeframe, both IL1α and CytoD led to a significant increase in **Ň** (fluid-like rheology), substantial drop in **S** (rounding of the nucleus), and modest but perceivable reductions in **R** (sDL-to-nucleus distance). Other factors (**Ĝ**_**u**_, 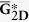, **Ĝ**_**v**_, **K** and **Ω**) showed distinct behaviors between treatments: on the one hand, IL1α induction increased anisotropy **K**, as a result of reduced **Ĝ**_**v**_, while exhibiting losses in **Ω**; on the other hand, CytoD treatments induced significant changes in hASCs for 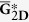, **K** and **R** (Fig. 4b).

It is worth noting that observations from this experiment after IL1α treatment matched those observations from Experiment I also under IL1α induction; in contrast, results after CytoD-induced remodeling were contrary to the effects observed in Experiment II in which hASCs experienced equal treatment conditions but while on μPP-constrained substrates. Given that this discrepancy for CytoD effects corresponded with distinct cell morphology conditions, we assessed whether additional differences in pnCSK mechanics observed after CytoD treatments in hASCs with unconstrained morphology would lead to discriminant power not seen in the case of μPP-constrained morphologies (see Experiment II for details). Indeed, multivariate profiling of cytokine effects on hASCs with unconstrained morphologies supported that expectation, as shown by areas under the ROC curve greater than 95% overall (Fig. 4c).

### Multivariate states of pnCSK mechanics across different human stem cells (Experiment IV)

To examine whether different stem cell types with different degrees of multipotency exhibit mechanical distinctions, we compared the mechanical profiles of hASCs, bone marrow-derived hMSCs and skin fibroblast-derived hiPSCs (Fig. 5a)–each expanded in monolayer under stem cell type-specific nondifferentiation conditions *in vitro* (see Methods section)–by inspecting their pnCSK microrheology and morphological features. We found that gains in sDL anisotropy **K**, fluidity **Ň**, nuclear shape **S**, distance from the nucleus **R** and losses in pitch angle from the nuclear major axis **Ω** were correlated with different types of human stem cells in different combinations. Interestingly, different stem cell types were indistinguishable from each other by the magnitudes of rheological properties, i.e. two-dimensional flow consistency index 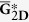 and either radial or angular shear moduli **Ĝ**_**u**_ and **Ĝ**_**v**_ (Fig. 5b).

Next, we asked whether each stem cell phenotype displayed characteristic mechanical signatures. Indeed, multivariate profiling of stem cells by discriminant analysis of pnCSK mechanics (Fig. 5c) showed high discriminant power among human stem cells, with areas under the ROC curve at about 96% in hASCs and hMSCs alike and above 99.5% in hiPSCs.

### Nondimensional maps of pnCSK states from diverse multipotent cell types

Two striking features exhibited by the entire pnCSK nondimensional spectrum of observations from all experiments are: a) collection of mechanical and structural properties within strong nonlinear correlations; and b) a finite range of bead distances from the nucleus **Θ** that converges to a fixed upper limit (Fig. 6a). These characteristics suggested that nondimensional pnCSK profiles are states of a cytoskeletal system that follows conserved mechanical principles in many cell types.

With the assumption that the mechanical behavior of the pnCSK obeys a conservative relation prevailing in all phenotypes, we can infer a baseline state of pnCSK mechanics coupled with nuclear shape and sDL localization; that baseline pnCSK state can be approximated by the distribution of observed pnCSK states after inducing cytoskeletal remodeling under various conditions. Such an experimental design resembles our experiments on hASCs (total number of cells [beads]: *N* = 163 [424] from Experiments I–III) that exhibit a reference pnCSK state at the median nuclear shape **S**_1/2_ (nuclear aspect ratio Φ_1/2_ = 1.6) for the ensemble dataset with: a) **Θ**_max_ < 3.1; b) tan(θ_*B*_)_1/2_ = 0.7; c) radially dominant **K**_1/2_ ≈ 31 [≪**K**≫ ≈ 50]; and d) elastically biased **ň**_1/2_ = 0.48 (Fig. 6a). In other words, at its basal state, the cell nucleus is about 60% longer than it is wide and is coupled to a pnCSK that extends less than 2 radii away from the nuclear perimeter, exhibits subdiffusive properties at perinuclear regions about 35° from the major nuclear axis, is highly anisotropic and stiffer in the radial direction, and slightly more solid- than fluid-like in response to stress.

### Empirical relations between pnCSK states and nuclear shape

Our experiments with stem cell phenotypes displayed counterbalancing responses among pnCSK rheology, nuclear shape and sDL spatial heterogeneity. To determine a quantitative relationship between these variables, we fit an empirical mixture model by recursive Bayesian inferencing (Fig. 6b top) where **S**^*a*^∙*b*^**Θ**^∙**Ω**^ln(**Ω**)−*c*^ = **K**^*d*^ (a = 0.57, b = 3.31, c = 2.49, d = 1.56; *R*^*2*^ = 0.89, *p* < 0.0001). As this model quantifies, our data suggests that both: a) the mechanical properties of the pnCSK reflect a conserved correspondence between rheological and geometric factors in the subdiffusive regions of the pnCSK; and b) nuclear shape and anisotropy are linked through sDL localization. Also, we confirmed a significant correlation between reference viscoelasticity **G″/G′** at **ƒ**_P_ = 1 Hz and fluidity **Ň**, which is expected of materials with power-law rheology (Fig. 6b bottom).

Here we used an elliptical coordinate basis that was mathematically prescribed by the shape of the nucleus to characterize pnCSK mechanics in each stem cell; using this approach, we clearly show that heterogeneous rheological properties in the pnCSK correlate inversely with nuclear shape and, surprisingly, also follow the same empirical fit in all cell types we investigated when explored in a unifying nondimensional space. These results are consistent with recent evidence linking the contractile history of the cytoskeleton with chromatin state and nuclear architecture^26^, suggesting that structural connections between nucleus and cytoskeleton must exhibit reciprocal mechanical properties. The corollary to such findings is that the mechanical properties of one (e.g. the cytoskeleton) may be predicted from the other (the nucleus).

In that context, and given the nondimensional relations between pnCSK mechanics and nuclear shape hold across different stem cells with different nuclei, our findings highlight one relevant question we can investigate: are such mathematical relations among mechanical or spatial cytoplasmic properties the same from stem cell to stem cell, except they appear dissimilar when their nuclei have different shapes? We approached this possibility by testing whether the localization and rheology of sDL in the pnCSK are predicted by the nuclear geometry. We found through cumulative frequency analysis that the distributions of **Ω**, **R** and **Ň**–i.e. position and fluidity of sDL in the pnCSK–were well modeled by fixed lognormal distributions whose parameters are univariate functions of nuclear shape **S** (parametric survival analyses *p* < 0.001; see Fig. 6c and Table 1). Since the rest of nondimensional parameters (Table 1) are predicted by different combinations of **Ω**, **R** and **Ň** and **S**, and each is a function of **S** itself, the nondimensional factors in our model can be estimated by only measuring the shape of the nucleus–in other words, pnCSK mechanics are numerically tractable by the nuclear shape. This is a result with powerful implications: it suggests that anisotropy and heterogeneity in cytoskeletal organization are interconnected mechanical properties founded on a cytoskeletal subdomain around the cell nucleus, tied to the structure of the nucleus, and quantifiable from its shape.

## Discussion

The findings of this study present a multivariable reduction strategy that lends a consistent frame of reference to characterize perinuclear mechanical properties of adherent cells. With this approach, we reveal that the nuclear shape of stem cells scales the relations among orthotropic cytoplasmic mechanics near the nucleus. Furthermore, these relations comprise distinctive mechanical signatures for different stem cell types, all of which are numerically tractable from the geometry of their stem cell nucleus. In short, we demonstrate that mechanical and morphological profiles, just as genetic and epigenetic ones, are powerful discriminants of stem cell phenotypes. In particular, we found that hASCs and hMSCs, both of which have lower differentiation potential than hiPSCs, are more elastic and anisotropic, have more elongated nuclear profiles, and host sDLs further away from the nucleus and closer to the nuclear major axis than hiPSCs (Fig. 5b). Such differences in the distribution of sDL and structural bias of the nucleus and its surroundings suggest that cytoplasmic mechanics in perinuclear regions can distinguish different stem cell types.

Our approach consistently unveiled symmetries within the cytoplasm of stem cells. The key is to obtain a single multidimensional map, one that harbors the individuality in the shape of each cell tested and, at the same time, collects heterogeneous cytoplasmic mechanics from many cells at once. To do this, we devised an elliptical coordinate system based on the shape of the nucleus in each cell and assumed orthotropic mechanics. From a geometric perspective, this strategy allows the coordinate systems from all cells, after geometric normalization with respect to their individual nuclear ellipses, to converge onto a nucleus-normalized canvas with all nuclear perimeters superimposed along the unit circle (Fig. 1c; see Methods and Supplementary Discussion for further details). From a rheological perspective, implementing an orthotropic model offers the ability to capture cytoplasmic anisotropic mechanics that, given an elliptical and curvilinear coordinate system, are unique to each bead in each cell because: a) they are characteristic of the original location of each bead with respect to the nucleus; b) they can only be traced back to the tested cell if the shape of its nucleus is known; and c) they estimate cytoskeletal mechanics from beads that, *when measured*, exhibited a window of subdiffusive-like motion inside the cytoplasm of stem cells–one that may or may not occur at any other time even for that specific cytoplasmic bead.

On a broad scope, our findings imply that experimental designs using cell-independent coordinates, planar rheology, or single-frequency tests may be particularly challenged in determining mechanical signatures of differentiation conveyed by cytoskeletal networks inside stem cells. Experimentally, an elliptically-based measure of pnCSK rheology may provide greater statistical power to resolve site-specific differences in properties as compared to Cartesian-based schemes. For example, displacements in Cartesian **x**- and **y**-directions of beads near the tips of the nucleus are downweighed by those in beads by the flanks if cytoplasmic mechanics are orthotropic, hence inflating the required sample size to observe statistically significant anisotropic effects; for those same beads, such muting effect is less pronounced in an elliptical system in which orthotropic bias is statistically additive - thereby reducing variance, enhancing statistical power and requiring less samples to match the statistical significance observed in Cartesian coordinates (see Fig. 1b).

Through these studies, we also concluded that the mechanics of perinuclear regions in stem cells are influenced by both biochemical and morphological input. The structural evidence acquired from all experiments in this work–including experiments testing the effects of biochemical inducers of cytoskeletal remodeling in hASCs with unconstrained or micropatterned morphologies–show that: a) cytokines alter the spatial distribution of sDL, usually accompanied by changes in the nuclear shape; b) redistribution of sDL in different cell morphologies is accompanied by changes in mechanical anisotropy; and c) pnCSK networks are sensitive to F-actin disruption, yet establish a steady mechanical profile within minutes that is conserved despite ongoing peripheral F-actin depolymerization (Figs 2, 3, 4 and Supporting Table S2; see Supplementary Information for further discussion). Throughout all these exchanges, perinuclear mesh networks can remodel nuclear shape and alter mechanical properties of cells dynamically, suggesting that the pnCSK acts as an endogenous unit in stem cells. We thus conclude that this approach for nondimensionalization of pnCSK mechanics, which quantifies how cells jointly offset their mechanical phenotype and nuclear architecture, portrays a functional map of biophysical states in pnCSK mechanics. Such information provides a means of quantifying the structural properties of the pnCSK in a consistent manner, and holds potential for screening cells based exclusively on pnCSK mechanics to distinguish multipotent phenotypes (Fig. 5c).

We used endocytic delivery of beads in our experiments. One advantage of this approach is that it yields more cytoplasmic beads for testing than biolistic delivery, another traditional method of bead delivery; one drawback is that endocytic delivery also returns more beads with driven or nonthermal motion–like the one observed when motor proteins shuttle endosomes^44^,^46^,^47^ or in regions with large energetic fluctuations inside cells^48^,^49^,^51^. Those movements do not fit GSER theory, which describes random thermal displacements of a spherical particle counteracted by its own drag inside a passive medium^45^. Beads with thermally-driven motion tracked across a range of different time windows τ exhibit a mean squared displacement ≪Δr^2^(τ)≫ with a log-scale slope α(τ) ≡ ∂[log (≪Δr^2^(τ)≫)]/∂[log (τ)] ≤1, where α(τ) = 0 for a purely elastic medium (e.g. no motion inside a solid), α(τ) = 1 for a purely viscous medium (e.g. free diffusion inside a Newtonian fluid), and 0 < α(τ) < 1 for a viscoelastic medium. In contrast, spherical probes with enthalpic or persistent motion exhibit α(τ) > 1, which breaks down the GSER model and is particularly evident when tested at large τ. Nevertheless, we still chose endocytic delivery because the benefits of biolistic techniques diminish with increasing probe size–for example, rheological estimates are comparable between either method when using 1-μm beads like ours^40^–and because biolistic methods also yield superdiffusive beads that must be filtered out using similar α(*t*)-based criteria^50^.

We recognize that the medium surrounding endocytosed beads inside live cells is not passive: it is composed of bioactive monomers, fibers, hydrolyzable molecules, shuttling proteins, metallic ions and catalytic enzymes in a mixture held together by the cytoskeleton^40^,^41^,^42^,^43^,^44^,^45^,^46^,^47^,^48^,^49^,^50^,^51^. All these are heterogeneously distributed inside the cytoplasm and, as a result, lead to locally varying cytoskeletal turnover rates ranging from a few seconds in lamellipodia to several minutes in stress fibers; these local rates depend on the local pools and kinetics of cytoskeletal reactants and can change over time^56^,^57^,^58^. So how can GSER theory apply to bead displacements in this context? Because cytoskeletal rearrangements fluctuate between Brownian and nonthermal windows of energy transfer, and are spatially heterogeneous. Then, GSER-based cytoplasmic rheology would be justified so long as measurements come from bead displacements captured within short intervals relative to local cytoskeletal turnover rates (e.g. seconds instead of minutes near stress fibers^56^,^57^,^58^) and fitting a Brownian profile. Therefore, in performing our analyses, we discriminated against: a) single beads within large vesicles, which exhibit motion dominated by each vesicle’s aqueous environment instead of cytoskeletal deflections; b) clusters of beads that, together, do not exhibit a spherical drag profile; and c) beads with nonthermal motion. We collected brightfield or epifluorescence microscopy images from each tested cell before recording. To filter against (a) and (b), we did not track bead clusters nor bead “singlets” inside large vesicles–the latter easily identified by light scattering from membranes of vesicles that appear separated from the contour of beads inside them. Last, to discriminate against (c), beads with α(τ) >1 were discarded from our data sets in subsequent analyses. We believe those conditions are why we captured patterns in cytoplasmic rheology: our data represents cytoplasmic beads tightly enveloped in F-actin, tracked during a window of subdiffusive motion, and distributed heterogeneously inside the cytoplasm–thus our reference to their locations as subdiffusive loci (sDL).

Anisotropic cytoplasmic mechanics expected from filamentous polymers, such as the cytoskeleton, have been previously reported based on Cartesian coordinate systems^46^,^48^. Our approach also revealed that anisotropy is locally varying within the cytoskeleton, independent of lagtime, and well described by a power-law; these features are consistent with a contractile active gel mechanically coupled to a deformable but relatively stiffer core^42^. Yet, here we also provide a unifying frame of reference for cells with different geometries that links radial distance and angle from the nucleus, nuclear shape, and degree of perinuclear anisotropy in the cytoplasm through a constitutive empirical relation; notably, this relation was conserved in all stem cell populations we tested. These findings concur with a polymeric cytoskeletal mesh model enhanced by radially interwoven filaments, an interpretation experimentally consistent with perinuclear actin cables wrapping around the nucleus and connected by transmembrane actin-associated nuclear (TAN) lines^29^. From a structural perspective, these physical features are reminiscent of a hydrostat^59^,^60^, only in this case applied at a microscopic scale on the stem cell nucleus and displaying phenotype-specific mechanical states. Thus, we posit that the pnCSK is a fiber-enhanced mechanical system that bridges physical interactions between the cytoplasm and nucleus of human stem cells in a phenotype-specific manner.

To better appreciate the relevance of implementing our model in analysis of PTM data from intracellular mechanics experiments, the elliptical map of pnCSK mechanics can be transformed back into a rectangular coordinate system. From this perspective, it is possible to extract an empirical relation that describes the behavior of the “nucleocytoskeleton” as a coupled unit, in which relocation of perinuclear subdiffusive sites and nuclear shape changes are reciprocal. This empirical relation can be used to generate hypotheses in stem cell mechanics that may explain, for example, how physical interactions between the nucleus and pnCSK evolve under loading, and provides testable predictions in cell morphology and cytoplasmic organization. In fact, when we performed vectorial analyses of infinitesimal shear stress-strain ratios and fit empirical relations between sDL localization and nuclear shape (please refer to Supplement for at-length discussions), our observations predicted that pnCSK deformations were mechanically coupled to changes in the nuclear shape in a systematic manner. For example, we find that local strain at sDL in the pnCSK of rounded cells under stress is prone to elongating the nucleus, whereas a distended pnCSK in elongated cells tends to pull inwards and round up the nucleus (Supporting Fig. S2); both these behaviors have been observed directly^3^,^26^. In this sense, our analysis suggests that the mechanically coupled pnCSK and nucleus constitute a single mechanical complex, one in which a nucleus “core” acts in combination with an enveloping and more deformable pnCSK, and that exhibits: a) mechanical equilibrium in a pre-stressed pnCSK state; b) uniform tugging by the pnCSK on the nucleus along stress-bearing sites; and c) stress-bearing sites found at about 30.4° < θ < 56.6° from the major nuclear axis when in equilibrium. Furthermore, sDL in this mechanical model are candidate stress-bearing sites along which mechanical stress reaches the nucleus (Supporting Fig. S2).

Looking ahead, we offer a practical role for the nuclear shape as a quantifiable indicator of transient cytoplasmic mechanics in stem cells, particularly as an accessible measurement to estimate pnCSK properties dynamically. Here we have shown that cytokines alter the spatial distribution of sDL, usually accompanied by changes in the nuclear shape, and that redistribution of sDL in different cell morphologies is counterbalanced by changes in mechanical anisotropy. Additionally, our data show pnCSK networks are sensitive to F-actin disruption, yet establish a steady mechanical profile within minutes that is conserved despite ongoing peripheral F-actin degradation (Supporting Table S2 and Figs 2, 3, 4) suggesting that the nucleus and its nearby surroundings resisted the structural effects of induced actin depolymerization better than the outer regions of the cytoplasm where cytoskeletal integrity was grossly affected. It is thus likely that the pnCSK is an impervious architecture that remodels its structural organization to sustain physical stress, compensate for a structurally weakened “outer” cytoskeleton, and encase the stem cell nucleus, thus lending it mechanical protection. In that role, the perinuclear subdomain of the cell cytoskeleton would in effect behave as a structural liaison “buffering” mechanical input on cells down its way to the nucleus. This is not to say that this perinuclear domain is not part of the cytoskeleton, only that its mechanical properties are different from the cytoskeleton further out. We suspect this may be due to differences in how cytoskeletal networks are assembled near and far from the nucleus, although the makeup of the perinuclear region may also play a role.

The nucleus and cytoskeleton are interactive elements which harbor tractable information about the mechanical state inside cells. In stem cells, there are many biological mechanisms involving the cytoskeleton that show strong correlations between nuclear shape and cell mechanics, such as cell spreading^3^,^26^. With our work, we not only confirm that nuclear shape, cytoplasmic rheology and anisotropy are reciprocal; we also find that: a) those variables converge to a master nondimensional mechanics map; b) stem cell types differ mainly on how they counterbalance those properties; and c) nondimensional cytoplasmic mechanics in stem cells can be estimated from their nuclear shape (Fig. 6). We also observed how pnCSK mechanics can stabilize quickly even as peripheral cytoskeletal networks continue to degrade (Supporting Table S2 and Figs 2, 3, 4). These observations suggest that perinuclear networks maintain an ability to exchange mechanical information with the nucleus, even during biological processes that elicit widespread structural remodeling inside and out of cells–such as stem cell differentiation. Altogether, we conclude that the stem cell pnCSK behaves in association with the nucleus as a mechanically distinct endogenous unit.

In summary, we confirm a specialized role for a finite perinuclear domain, the pnCSK, which interacts with the nuclear structure to coordinate overall cell mechanics. Moreover, we present evidence of intracellular form-function relations that quantify the homeostatic mechanical counterbalance between nuclear shape and pnCSK architecture under a range of exogenously induced phenotypes. Finally, we also show that the shape of the cell nucleus in human stem cells: a) is sufficient to map both the spatial distribution and scale of intensive rheological properties within subdiffusive domains of the pnCSK; and b) reflects distinctive mechanical states within a common parametric map that is representative of multiple cell phenotypes. This work advances a new approach to examine intracellular mechanics in stem cells from different lineages, with distinct biophysical properties, or under various forms of metabolic regulation as functions of mechanical pnCSK states, all of which lie within probabilistic distributions predicted by the shape of the nucleus and, therefore, measurable by standard microscopy alone.

## Methods

### Use of experimental animals and human subjects

No vertebrate animals or human subjects were used in this study. Human cells were used from previously isolated cell lots taken from de-identified donors in accordance with an exemption from IRB review, and are not considered human subjects.

All human donor-derived cells later used in our experiments (human adipose- and bone marrow-derived mesenchymal stem cells) originated from previously screened and long-term cryopreserved individual lots based on proliferative and multilineage differentiation capacity when grown in culture medium containing lot-selected fetal bovine serum (FBS). To minimize the potential effects of donor bias in subsequent experiments for each cell type, three from all available individual donor-specific lots were selected and pooled into multi-donor superlots, as described next.

### Human adipose-derived mesenchymal stem cell (hASC) culture

Stocks of hASCs were derived from discarded and de-identified subcutaneous liposuction waste adipose tissue from nonsmoking, nondiabetic Caucasian donors following established protocols^61^. Freshly isolated hASCs from three donors were individually expanded in standard culture conditions (37 °C and 5% CO_2_), pooled into a single superlot after a single passage in equal numbers (SL-JF09) and used in all subsequent studies. Cells were cultured in either stromal media during experiments (STM: DMEM/F12 [Gibco] +10% heat-inactivated FBS [HI-FBS, Gibco]) or hASC expansion media for banking or serial expansion (DMEM/F12 [Gibco] +10% lot-selected FBS [Atlas Biologicals] +0.25 ng/mL human platelet-derived transforming growth factor beta 1 [hPD-TGFβ1, R&D Systems] +5 ng/mL recombinant human epidermal growth factor [rh-EGF, Roche] +1 ng/mL recombinant human basic fibroblast growth factor [rh-bFGF, Roche]).

### Human bone marrow-derived mesenchymal stem cell (hMSC) culture

Stocks of hMSCs were isolated following established protocols^62^ from discarded and de-identified waste bone marrow tissue from adult transplant donors at Duke University Medical Center in concordance with an Institutional Review Board exemption. Adherent cells from three donors were expanded individually for two passages in standard culture conditions (37 °C and 5% CO_2_), pooled in equal numbers into a single superlot and used in all subsequent studies. Cells were cultured in either STM during experiments or hMSC expansion media for banking or serial expansion (DMEM-LG [Gibco] +10% lot-selected FBS [Hyclone] +1 ng/mL rh-bFGF [Roche]).

### Human induced pluripotent stem cell (hiPSC) culture

A human induced pluripotent stem cell (hiPSC) clonal line derived from human fibroblasts (CCD-1079Sk; ATCC cat. no. CRL-2097, kindly donated by Dr. Kam W. Leong, Columbia University) was cultured in adherent culture plates coated with truncated recombinant human vitronectin at 5 μg/cm^2^ (VTN-N, Invitrogen) and fed with defined xeno-free Essential 8™ medium (E8, Gibco) as recommended elsewhere^63^.

### Fibronectin coating of culture dishes

To enhance hASC and hMSC attachment through the duration of live microscopy experiments, either Mat-Tek low-profile glass-bottom dishes (50-mm × 14-mm well, #1.5 cover slip) or 42-mm #1.5 round cover slips were coated under sterile conditions with fibronectin (FN, Sigma-Aldrich) at 5 μg/cm^2^ by incubation for 1 hr in culture conditions with 300 μl of gently dissolved 20 μg/ml FN solution in 1X phosphate buffered saline (PBS pH 7.4, Gibco) containing 0.1% F-127 pluronic acid, followed by 5-min incubation at room temperature with 3 ml of 2 M NaCl in H_2_O and blocking with 1.5 ml of 1% heat-inactivated bovine serum albumin (BSA, Gibco) solution in PBS ph 7.4. Single dishes were washed 3 times with 3 ml PBS between steps. Coated dishes remained hydrated in 3 ml PBS pH 7.4, parafilmed individually under sterile conditions, and stored at 4 °C until use within 1–2 days.

### Microphotopatterning (μPP)

Cell-adhesive patterns were created within a non-fouling hydrogel layer as described elsewhere^53^. Glass-bottomed cover dishes (MatTek Corp) were amino-silanated (1% (3-aminopropyl)trimethoxysilane, Sigma), activated with 0.5% glutaraldehyde, and spin-coated with polyvinyl alcohol (Sigma, 5.6% w/v in 0.2N HCl) to create a thin (~150 nm thick) hydrogel layer that resists protein adsorption and cell adhesion (stable for >1 month in culture). Next, μPPs (64/dish) photoablated within the gel layer using a two-photon microscope (Olympus FV1000, 25 × 1.05NA objective, Ex: 725nm) were coated with fibronectin (4–5 μg/sq.cm, Sigma-Aldrich) followed by blocking with 1% heat-denatured BSA (Life Technologies) to promote cell adhesion. Diverse rectangular patterns were tested using hASCs to select a single rectangular geometry; a pre-screened 15 × 70 μPP geometry (15-μm × 70-μm rectangle) that exhibited single-cell attachment and cell morphology control was chosen.

### EDAC-based fluorophore conjugation of carboxyl-functionalized magnetic beads

Superparamagnetic, low-coercivity 1-μm beads with carboxylic acid surface functionalization (Dynabeads® MyOne™ Carboxylic Acid, Life Technologies) were cross-linked to either AlexaFluor 647-conjugated 10-kDa anionic dextran molecules (AF647) or microinjection-grade AlexaFluor 568 (AF568) by carbodiimide-based crosslinking. Briefly, 1 mg of MyOne-COOH beads were incubated with 50 μg of AF647 with shaking for 30 min at room temperature, followed by addition of 0.3 mg of 1-Ethyl-3-[3-dimethylaminopropyl]carbodiimide hydrochloride (EDAC) in a final 100-μl total reaction volume. All reagents were delivered in solution with 25 mM MES pH 6.0 buffer. The final cross-linking reaction volume was incubated overnight at 4 °C with shaking, followed by 2-min magnetic isolation of beads and 5 subsequent washes with 0.5 ml of 50 mM Tris Buffer pH 7.4 to quench remaining carboxylic reactive ends. After the last 2-min magnetic isolation from quenching supernatant, the fluorescently labeled (MyOne-AF568/647) beads were resuspended at 10 mg/ml in Opti-MEM +0.02% NaN_3_ for storage. Diluted MyOne-AF568/647 aliquots at 1 mg/ml in H_2_O were also prepared from stocks for continued use and had their fluorescence signal confirmed by confocal microscopy on coverglass before experiments.

### Bead lipofection, replating and transient actin-GFP transfection of hASC cells

MyOne-AF568/647 beads were introduced by lipofection into the cytoplasm of stationary phase, near-confluent (80–90%) hASC SL-JF09 cells on 2 × 35-mm plastic culture dishes at passage 3. Following the manufacturer’s instructions, 500 μl of Opti-MEM containing 4 μg of MyOne-AF568/647 beads and 10 μl of Lipofectamine 2000 (Life Technologies) were incubated for 30 min at room temperature per dish, then added the lipofection mix to each 35-mm hASC culture dish with 2 ml of antibiotic-free stromal media. Then, cells were incubated for 2–3 hours in culture conditions to allow bead endocytosis to occur. Cells from individual dishes were then washed with 1X DPBS, lifted and collected by trypsinization for 10 min in culture conditions with 0.05%Trypsin/EDTA solution, pelleted at 300 × g for 5 min and resuspended in 0.6 ml of cold expansion media. Bead-lipofected hASCs, or hASCs(+), were isolated from each vial by subsequent parallel magnetic isolations. hASCs(+) from the second magnetic isolations (8,000–10,000 per 35-mm dish) were pooled by resuspension in 0.8–1.0 ml of expansion media. To induce transient actin-GFP fusion protein expression, a commercially available actin-fluorophore fusion baculovirus expression system was inoculated (CellLight® Actin-GFP/RFP or mitochondrial PDHA1-RFP BacMam 2.0, Life Technologies) by adding 8 μl, or about 50 particles per cell (PPC), into the hASC(+) pooled cell suspension. Inoculated hASCs(+) were plated (passage 4) onto FN-coated substrates (above) at 250–300 μl per substrate for 16 hours to enhance attachment and transduction in culture. Expansion media was refreshed, and attached actin-GFP hASCs(+) were allowed to accumulate actin-GFP fusion protein expression before testing for 1–2 days post-plating.

### Validation of experimental setups for particle-tracking microrheology (PTM)

Bead motion was recorded using brightfield or phase contrast microscopy. In total, we implemented four different testing configurations (Supporting Table S1). Each configuration was validated by Brownian motion analyses of bead displacements in Newtonian sucrose solutions at room temperature (2.0 and 2.5 M, ~10^6^ beads per ml, recorded for 30–60 s at 100–120 fps) using two-dimensional GSER. Bead positions were approximated from Gaussian diffraction profiles of beads; subsequently, bead displacements were calculated and discriminated against a sub-pixel resolution threshold of 1/20th of a pixel. This validation protocol, performed before each test, confirmed reproducibility among all implemented PTM testing configurations, consistently exhibiting a constant viscosity two-decade frequency span between 0.1 Hz–10 Hz with phase lag error <5.7° (damping error G”/G’ <10%) in our sucrose calibration solutions. All bead selection, post-processing, and rheological analyses were based on rheological measurements within this two-decade frequency range of resolvable viscoelasticity, which also corresponds to testable time windows with statistically robust random walk measurements (0.1 s < τ < 10 s).

### Bead tracking data post-processing

Mathematical principles of PTM data analysis in orthogonal coordinate systems have been extensively described elsewhere^44^,^45^. In summary, positions of intracellular beads were acquired from brightfield microscopy videos (15–60 s) using tracking analysis software and data post-processing script libraries developed by the Superfine group at the Center for Computer Integrated Systems for Microscopy and Manipulation (CISMM) at UNC Chapel Hill. Before rheological analyses, position 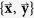-coordinates of beads within individual cells were translated, rotated and transformed with respect to the centroid and major axis of the best-fit ellipse to the thresholded fluorescence image of the cell nucleus into a quadrant-symmetric elliptical *uv*-coordinate system with orthonormal covariant unit basis. The transformed data was processed to determine the slope 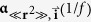 of the (log-scale) time-dependent mean-square displacement ≪Δr^2^(τ)≫ and 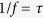 for 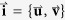 in each bead, then parsed into two distinctive groups discriminated with respect to their mathematical random walk classification as defined by their maximum 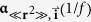 across the measured 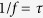 range: subdiffusive motion for 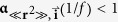; and superdiffusive motion for 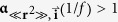. Post-processed position data in elliptical coordinates from subdiffusive beads were individually analyzed and then collected for statistical analyses through an automated custom-scripted analysis pipeline in MATLAB (The MathWorks, Inc., Natick, MA, USA). The automated analysis routine was validated in advance by confirming agreement between Cartesian (source-scripted, CISMM) and elliptical-transformed (custom-scripted, ORL) analyses outcomes between two-dimensional ≪Δr^2^(τ)≫ measurements.

### Qualitative time-lapse confocal microscopy of cytoskeletal actin

To obtain *a priori* qualitative confirmation of short-term cytoskeletal effects on hASCs during cytokine stimulation at physiologic levels in joint disease^64^, actin-GFP hASCs for Experiment III (equipment described in Supporting Table S1) were plated in static culture onto FN-coated MatTek dishes, stained for 30 min with Hoechst 33342 live nuclear dye (HO342), and set up in a humidified automated heated stage for laser scanning confocal microscopy. After 30–60 min of thermal stabilization to 37 °C, media for actin-GFP/PDHA1-RFP hASCs was substituted with stromal media, cells with positive fusion protein expression were defined and recorded for automated stage repositioning (N = 10 per experiment), and culture was supplemented with either 10 ng/ml rh-IL1α (R&D Systems), 10 ng/ml hPD-TGFβ1 (R&D Systems), or none (control condition). Finally, continuously repeated single-plane time-lapsed confocal microscopy was performed for 1 hr at approximately 6 min per iteration (Experiment III setup, as described in Supporting Table S1); single focal planes in each cell were determined before treatment to correspond with maximal HO342 epifluorescence area (i.e. the central transverse plane of the cell nucleus).

### PTM and fluorescence (PTM+F) microscopy of cytoskeletal remodeling

As an initial reference study of the spectrum of rheological and structural cytoskeletal dynamics following exogenous treatment with either anabolic or catabolic cytokines, MyOne-AF568 hASCs(+) for Experiments I and II (equipment described in Supporting Table S1) were inoculated with viral actin-GFP fusion protein reagent at 50 PPC in expansion media as described previously (300 μl per cover slip), replated onto FN-coated MatTek dishes to obtain either unconstrained or μPP-directed hASC morphology (Experiment I or II, respectively), and stained for 30 min with HO342. Prior to testing, actin-GFP|MyOne-AF568 hASCs(+) were washed briefly with 1X PBS pH 7.4 and incubated for 1 hr at 37 °C in static culture with treatment-specific media consisting of stromal media supplemented with either 10 ng/ml recombinant human IL1α (rh-IL1α, catabolic), 10 ng/ml human platelet-derived TGFβ1 (hPD-TGFβ1, anabolic), or none. Following treatment interval, cultured cover slips were pushed off MatTek dishes with a single disposable 8-mm biopsy punch per dish, taped onto the microscope stage and replenished with treatment-specific media. Our testing protocol consisted of DAPI, FITC and Texas Red filtered epifluorescence image acquisition immediately followed by 30-sec brightfield video acquisition at 120 fps of sequentially selected actin-GFP|MyOne-AF568 hASCs(+). Cells for data acquisition were selected on the basis of moderate actin-GFP expression in stress fibers and tested as found. Full treatment-specific samples were tested within 1 hour after cover slip setup at room temperature. All experiments were performed within 1–2 days after plating onto FN-coated surfaces.

### PTM and confocal (PTM+C) microscopy of cytoskeletal remodeling

To study progressing short-term rheological and structural cytoskeletal dynamics after exogenous biochemical treatment, MyOne-AF647 hASCs(+) for Experiment IV (equipment described in Supporting Table S1) were inoculated with viral actin-RFP fusion protein reagent at 50 PPC, plated overnight in culture conditions onto FN-coated Ø 42-mm #1.5 cover slips with expansion media, and stained for 30 min with SYTO13 green fluorescent live nucleic acid dye before magnetic selection. Expansion media with transduction reagent was substituted with stromal media on the next day for at least 1 hr in culture conditions. Next, cultured cover slips were assembled into an open-lid, open-loop perfusion chamber with 2 ml of stromal media in a humidified automated heated stage for laser scanning confocal microscopy as described previously at ORL. After 30–60 min of thermal stabilization to 37 °C, actin-RFP|MyOne-AF647 hASCs(+) with moderate actin-GFP expression in stress fibers were selected (N = 10) and their locations were registered and prescribed to a confocal plane with maximal SYTO13 fluorescence area (i.e. the central transverse plane of the cell nucleus). Confocal microscopy and video acquisition (PTM+C) was then performed on prescribed actin-RFP|MyOne-AF647 hASCs(+) in stromal media as pre-treatment control. Stromal media in the perfusion chamber was fully replaced twice with treatment media (stromal media supplemented with either 10 ng/ml rh-IL1α, 0.5 μM Cytochalasin D or none) using synchronized input/output syringe pumps at 4 ml/min with 1-min delay between cycles. For post-treatment testing after complete media replacement, input/output perfusion rates were slowed down to 0.3 ml/min of treatment media for the next 2 hours to induce steady-state stimulation by continuous media refreshment. Finally, repeated single-plane time-lapsed confocal microscopy was performed on all prescribed cells at 30-min intervals (approximately 12 min per iteration) with PTM + C testing at 0 hr, 1 hr and 2 hours post-treatment. Our iterative PTM/C testing protocol consisted of double confocal imaging and 30-s brightfield video acquisition at 100 fps within a 1-min delay between images. In all cell iterations, confocal planes were manually fine-tuned after the first confocal image to correspond with the evolving nucleus in each prescribed cell, with MyOne-AF647 beads used as relative fiducial marks between imaging time points. Geometric descriptions for each prescribed cell used data from second confocal image, i.e. image at manually updated confocal planes.

### Statistical analysis

Sample sizes were determined *a priori* by power analyses using log-transformed preliminary data to detect differences at standardized effect size *δ/σ* ≥ 1.5, significance level *α* ≤ 0.05 and statistical power of at least 90% (Bonferroni approximation) for multi-factor, matched-pair, nested and/or repeated measures ANOVA testing formats. Sample normality of log-transformed parameters (color) was confirmed in each experiment by both Shapiro-Wilk (log-transformed data) and Kolmogorov (linear data) testing. For repeated measures ANOVA, Mauchly’s sphericity test was performed to introduce Greenhouse-Geisser (ε < 0.75) or Huynh-Feldt (ε > 0.75) significance test adjustments as needed. Significance levels in ANOVA tests are represented graphically as follows: *p < 0.05, **p < 0.01, ***p < 0.001 and ****p < 0.0001. *Post hoc* inferential significance tests within parameters were performed by Tukey-Kramer tests for mean differences (*α* < 0.05). Satisfactory statistical power (adjusted 90% level) was confirmed in all statistically significant *post hoc* tests; in addition, both the least significant sample number (LSN) and value (LSV) to 50% adjusted statistical power were corroborated. Equivalence between samples was determined by the two one-sided tests (TOST) method at a null difference level of 50% root-mean squared error (RMSE). Significant effect sizes between groups with respect to non-normally distributed variables were determined by cumulative frequency analyses via Cox Proportional Hazards models, following *a priori* confirmation of non-intersecting hazard functions in all cases. All statistical analyses were performed with JMP Pro 10 software (SAS, Cary, NC). Unless stated otherwise, error bars for experimental data depict mean ± standard error of the mean (s.e.m.) intervals. In each experiment, groups not sharing letters within a parameter are statistically significant. All experiments were performed at least in triplicate.

### Principal Component Analysis (PCA)-based variable reduction

Rheological data was modeled using a power-law model over a limited frequency span (0.2 Hz < **ƒ**_P_ < 5 Hz) flanked by low and high frequency regimes with statistically equivalent moduli in each (TOST equivalence testing). Specifically, 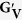 is the plateau storage modulus in the low-frequency domain 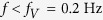 in which within-group equivalence 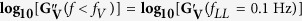 is true for all groups; conversely, 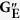 is the plateau loss modulus in the high-frequency domain 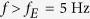 where 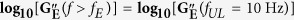 is true for all groups. Each groups’ equivalence domain was established by TOST equivalence tests for each shear moduli. With this methodology, we quantified four rheological descriptors per basis direction (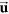 and 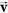): shear moduli 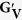, 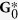, 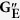; and flow behavior index 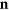. The overall 8-variable set was transformed by correlation-based PCA into orthogonal components, reduced by standard criteria (Kaiser/scree-plot) and re-parameterized by factor loading analyses (Varimax orthogonal rotation). Those reduced rheological factors **Ň** ≡ **ň**/(1 − **ň**) [where **ň **≡ ½∙(**n**_**u**_ + **n**_**v**_)], **Ĝ**_**u**_ and **Ĝ**_**v,**_in conjunction with geometric factors **S**, **Ω** and **Θ**, were used to identify outliers per group in each experiment by a multivariate jackknife distances method; subsequently, PCA-based variable reduction was re-inspected for adequacy and confirmed in all experiments by PCA on reduced rheological factors and confirmatory discriminant analysis (quadratic, unequal covariances, prior probabilities proportional to occurrence, model significance *p* ≤ 0.006 for all experiments).

### Discriminant analyses

Predictive machine-learning power is reported graphically via receiver operating characteristic (ROC) curves specific to each experimental dataset in terms of 2-factor canonical discriminant factor plots (central 50% density ellipses assume bivariate normal distributions within each independent group).

### Nonlinear fitting (NLF)

Frequentist models for nondimensional factors were calculated empirically by cumulative distribution analyses with respect to the nuclear shape **S** (parametric survival methods). Candidate multifactorial mixture models relating rheological and geometric responses were pre-screened via recursive Bayesian model testing (Box-Meyer method with equal prior probabilities, contamination coefficient *K* = 10, model reduction by sparsity-of-effects principle) and confirmed based on linear-fit regression between predicted and measured parameters (*R*^2^ with minimal residual sum of squares among pre-screened parsimonious models).

## Additional Information

**How to cite this article**: Lozoya, O. A. *et al*. Universally Conserved Relationships between Nuclear Shape and Cytoplasmic Mechanical Properties in Human Stem Cells. *Sci. Rep.* **6**, 23047; doi: 10.1038/srep23047 (2016).

## Supplementary Material

Supplementary Information

## Figures and Tables

**Table 1:** Nondimensional and predictive relations in pnCSK mechanics.

Nondimensional Factor	Functional Form	Parameterization
**S** [0.49 < **S** < 11.0]	**S** ≡ (1 − *ε*)/*ε ε *≡ [1 − (1/Φ)^2^]^1/2^; Φ ≡ *M*/*m* (*M*: major axis; *m*: minor axis)	**S** ≡ (Φ^2^ − 1) + Φ∙(Φ^2^−1)^1/2^
**Ω** ≡ tan(*v*_*B*_)	**Ω** ~ ln(μ_Ω_, σ_Ω_^2^)	μ_Ω_ ≡ ln(**Ω**_1/2_) = −0.19−0.27∙ln(**S**) σ_Ω_^2^ = 1.21 ≪**Ω**≫ = 1.83∙**Ω**_1/2_
**R** ≡ **Θ** − 1 [****R**** ≡ (*u*_*B*_ − *u*_*NE*_)/*u*_*NE*_]	***e***^**R**^ ~ ln(μ_**R**_, σ_**R**_^2^) → ****R**** (μ_**R**_, σ**R**^2^)	μ(*e*^R^) ≡ ln(*eR*_1/2_) = 0.35 + 0.12∙ln(**S**) σ_R_^2^ = (0.43 + 0.02∙S)^2^ 1.10∙******R******_**1/2**_ < ≪******R******≫ < 1.24∙**R**_**1/2**_
**Ň** ≡ **ň**/(1 − **ň**) [**ň** ≡ ½∙(**n**_**u**_ + **n**_**v**_); **ň** ≡ **Ň**/(**Ň** + 1)]	**Ň** ~ ln(μ_Ň_, σ_Ň_^2^)	μ_Ň_ ≡ ln(**Ň**_1/2_) = −0.20∙ln(S) σ_Ň_^2^ = 0.88 ≪ **Ň** ≫ = 1.55∙**Ň**_1/2_
**K** ≡ **Ĝ**_**u**_/**Ĝ**_**v**_; [**Ĝ**_**i**_ ≡ ( 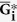 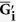 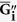 )^1/3^; **i** = {**u**,**v**}]	**S**^*a*^∙*b*^Θ^∙**Ω**^ln(Ω)-*c*^ = **K**^*d*^	*a* = 0.57, *b* = 3.31, *c* = 2.49, *d* = 1.56 (*R*^2^ = 0.89)
α(1/**ƒ** ∈ **ƒ**_**m**_) at **ƒ** = 1 Hz	tan(α∙^π^⁄_2_) ≡ **G″/G′**	tan[α∙(^π^⁄_2_)] ≡ **G″/G′** = [0.88∙**Ň**]^0.77^
