# Supplementary material for: Universally Conserved Relationships between Nuclear Shape and Cytoplasmic Mechanical Properties in Human Stem Cells
Source: Sci Rep. 2016 Mar 15;6:23047. doi: 10.1038/srep23047 (PMC4792134; doi:10.1038/srep23047)
Supplement: Supplementary Information [file srep23047-s1.pdf]

**SUPPLEMENTARY INFORMATION**

# Universally Conserved Relationships between Nuclear Shape and Cytoplasmic Mechanical Properties in Human Stem Cells

Oswaldo A. Lozoya[a], Christopher L. Gilchrist[b], Farshid Guilak[b,c,d]

[a]Epigenetics and Stem Cell Biology Laboratory, National Institute of Environmental Health Sciences, Research Triangle Park, NC 27709

[b]Department of Biomedical Engineering, Duke University, Durham, NC 27708

[c]Departments of Orthopaedic Surgery, Biomedical Engineering, and Developmental Biology, Washington University in St. Louis, St. Louis, MO 63110

[d]Shriners Hospitals for Children - St. Louis, St. Louis, MO 63110

**Corresponding Author:**

Farshid Guilak, Ph.D.
Shriners Hospitals for Children - St. Louis
4400 Clayton Ave.
St. Louis, MO 63110-1624, USA.
Email: guilak@wustl.edu

## Supplementary Discussion

**Nondimensional factors describe mechanical pnCSK properties.** The orthonormal basis components in curvilinear elliptical coordinates describe cytoplasmic bead position vectors $\mathbf{P}_B = u_B \cdot \mathbf{u} + v_B \cdot \mathbf{v}$ in terms of: a) radial unit vector $\mathbf{u}$ normal to elliptical locus $u_B$, confocal to the nuclear envelope ($u_{NE} \cdot \mathbf{u}$) and with reflected foci-to-foci length $2 \cdot M \cdot u_B$; and b) angular unit vector $\mathbf{v}$ normal to a hyperbola with a linear asymptote angle $v_B$ orthogonal and confocal with respect to the $u_B$ locus. Briefly, this transformation routine collects geometric information from each cell nucleus' best-fit ellipse – centroid position, nuclear aspect ratio $\mathbf{\Phi}=M/m$ [major and minor semiaxes $M$ and $m$, respectively] and interfocal distance $2 \cdot f_{NE}$ – to compute anisotropic bead displacements by: a) linear translation of each cell nucleus's centroid to origin at (0,0); b) rotation to superpose translated $x$- and $y$- axes onto $M$ and $m$ of elliptical nucleus with foci ($\pm f_{NE}$,0); c) numerical conversions of Cartesian bead locations ($x_B$,$y_B$) to curvilinear orthonormal elliptical coordinates ($u_B$,$v_B$) using transformation functions $x_B = f_{NE} \cdot \cosh(u_B) \cdot \cos(v_B)$, $y_B = f_{NE} \cdot \sinh(u_B) \cdot \sin(v_B)$ and covariant scale factors $h(u_B) \equiv h(\mathrm{v}_B) = f_{NE} \cdot [\sinh^2(u_B) + \sin^2(v_B)]^{1/2}$ with local orthonormal basis $\mathbf{i} = \{\mathbf{u},\mathbf{v}\}$. This geometric transformation routine was streamlined into the analytical pipeline to preclude user intervention bias.

Elliptical-based cytoplasmic rheological properties are characterized by the following properties: a) the elliptical and hyperbolic complex shear moduli $\mathbf{G_u^*}(\boldsymbol{f})$ and $\mathbf{G_v^*}(\boldsymbol{f})$ were frequency-dependent and distinct from each other ($p$<0.0001); b) anisotropic ratios among shear modulus components are equivalent in the range 0.1 Hz<$\boldsymbol{f}$<10 Hz and, i.e. $\mathbf{G_u^*}(\boldsymbol{f})/\mathbf{G_v^*}(\boldsymbol{f})=\mathbf{G_u'}(\boldsymbol{f})/\mathbf{G_v'}(\boldsymbol{f})=\mathbf{G_u''}(\boldsymbol{f})/\mathbf{G_v''}(\boldsymbol{f})$ (significance $p$>0.98, equivalence $p$<0.001); and c) amplitudes for all moduli and directions are proportional and display constant viscoelastic damping, since frequency dependence – analogous to $\alpha(t)$ in «$\Delta r^2(\tau)$» profiles – is indistinguishable among shear moduli $\mathbf{G_i^*}(\boldsymbol{f})$, $\mathbf{G_i''}(\boldsymbol{f})$, and $\mathbf{G_i'}(\boldsymbol{f})$ ($p$>0.86) and between $\mathbf{u}$ and $\mathbf{v}$ components ($p$>0.95). Consistent with these analyses, we found upon further inspection that pnCSK rheology (Figure 1d) exhibited power-law behaviors in all $\mathbf{G_i^*}(\boldsymbol{f})$, $\mathbf{G_i''}(\boldsymbol{f})$, and $\mathbf{G_i'}(\boldsymbol{f})$ for both $\mathbf{i}=\{\mathbf{u},\mathbf{v}\}$ in the range 0.2 Hz<$\boldsymbol{f}_P$<5 Hz and, in particular, statistically significant fits for $\mathbf{G_i^*}(\boldsymbol{f} \in \boldsymbol{f}_P)=\overline{\mathbf{G}}_\mathbf{i}^* \cdot \boldsymbol{f}^{\mathbf{n}_\mathbf{i}}$ with flow consistency and behavior indices $\overline{\mathbf{G}}_\mathbf{i}^*$ and $\mathbf{n_i}$, respectively (Ostwald-de Waele power-law model; per-bead least-squares fit: average $R^2$=0.90 $p$<0.001). Remarkably, $\mathbf{n_u}$ and $\mathbf{n_v}$ were statistically equivalent within groups in all experiments (Figure 1d; significance $p$>0.21, equivalence $p$<0.05), thus showing that rheological properties of the pnCSK have orthogonal shear moduli proportional to each other and which display equal frequency dependence at physiologically relevant time scales.

The power-law shear modulus $\mathbf{G_i^*}(f)=\mathbf{\bar{G}_i^*}\cdot f^{\mathbf{n_i}}$ was bounded statistically by two regions with a different frequency dependence: one low-frequency span $f_L<0.2$ Hz with constant $\mathbf{G_i'}$, i.e. $\mathbf{G_i'}(f\in f_L)=\mathbf{\bar{G}_i'}$ ($p>0.94$, equivalence $p<0.05$); and one high-frequency span $f_H>5$ Hz with constant or concave $\mathbf{G_i''}$ where $\mathbf{G_i''}(f\in f_H)\leq\mathbf{\bar{G}_i''}$ (average $p=0.44$, average equivalence $p=0.03$). These two subdomains are particularly interesting not only because they deviate from the power-law rheology model $\mathbf{G_i^*}(f)=\mathbf{\bar{G}_i^*}\cdot f^{\mathbf{n_i}}$ outside the range 0.2 Hz<$f_P$<5 Hz, but also because they diverge from the constant power-law phase angle $\boldsymbol{\delta_i}\equiv\tan^{-1}(\mathbf{G_i''}/\mathbf{G_i'})$ and predict viscoelastic transition frequencies at either end with steadily decreasing phase angles, i.e. viscous-to-elastic crossovers at slow and fast time scales with $\mathbf{G_i'}>\mathbf{G_i''}$.

We used principal component analyses to identify multivariate associations in logarithmic space among rheological parameters from cytoplasmic beads (Supporting Figure S1). Two unbounded nondimensional factors where determined: fluidity $\mathbf{\check{N}}\equiv\mathbf{\check{n}}/(1-\mathbf{\check{n}})$, based on the average flow behavior index $\mathbf{\check{n}}\equiv\frac{1}{2}\cdot(\mathbf{n_u}+\mathbf{n_v})$; and anisotropic ratio $\mathbf{K}\equiv\mathbf{\hat{G}_u}/\mathbf{\hat{G}_v}$, which relates the shear moduli geometric means $\mathbf{\hat{G}_i}\equiv[\mathbf{\bar{G}_i^*}\cdot\mathbf{\bar{G}_i'}\cdot\mathbf{\bar{G}_i''}]^{1/3}$ from the $\mathbf{i}=\{\mathbf{u},\mathbf{v}\}$ directions. We also normalized the positions of beads in cells by their nucleus' elliptical geometry to obtain a consistent geometric basis for comparison. Thus, given a cell nucleus with major and minor elliptical semiaxes $M$ and $m$, nuclear aspect ratio $\mathbf{\Phi}\equiv M/m$, nuclear eccentricity $\varepsilon\equiv[1-(1/\Phi)^2]^{1/2}$, and $u=u_{NE}$ at the nuclear envelope, we define a nondimensional map of bead positions in terms of the following factors: distance to bead $\mathbf{\Theta}\equiv u_B/u_{NE}$; angle to bead $\mathbf{\Omega}\equiv\tan(v_B)$; and c) nuclear shape $\mathbf{S}\equiv\varepsilon/(1-\varepsilon)$. This geometric description results in a polar map based on unit circles that corresponds to the nuclear envelope in each and all cells; hence, this strategy unifies the geometric description of bead positions under a single mapping distribution in nondimensional space (Figure 1c), which allows testing of spatial-dependent cytoplasmic rheology across cells with different nuclear shapes.

We performed three independent experiments to characterize hASC cytoskeletal mechanics in diverse environments (Table S1) and observed significant changes in mechanical properties of the pnCSK for all cases. In sum, we found that different mechanisms of exogenously-induced cytoskeletal reorganization express distinguishable mechanical signatures in hASCs, as summarized in Supporting Table S2 and Figures 2-4. From a spatial perspective, sDL maps were distinct between treatments and exhibited dense distributions, thus showing that sDLs in cells are nonrandom and reflect spatially heterogeneous pnCSK mechanical properties.

**Optimization of bead lipofection conditions in hASCs.** We selected a bead lipofection strategy for cells with no more than 8-12 beads each; we found that hASCs under these conditions maintained their viability and

*in vitro* adipogenic differentiation capacity. In effect, this meant that under our bead lipofection titer (roughly 1 μg of MyOne™ beads per 200,000 hASCs; see Methods section for details) beads entered the cytoplasm of some but not all cells in culture, in most cases appearing as "singlets" inside bead-containing cells. This cell fraction was easily purified after two rounds of magnetic selection (the first round designed to isolate non-absorbed beads) and could be passaged and grown in expansion media under normal conditions. We observed similar bead integration densities when bead lipofection was carried out on hMSCs or hiPSCs using the same protocol; in all cases, cell viability was maintained.

**Experimental considerations in PTM experiments with endocytic bead delivery.** Our experiments were based on endocytic delivery of cytoplasmic beads. In comparison, previous experiments on other stem cell models, namely human embryonic stem (hES) and human induced pluripotent stem (hiPS) stem cells, used beads impinged through biolistic delivery. It is unclear whether such discrepancy in experimental methodology lies behind the differing nature of their measurements where the discriminant factor between stem cell phenotypes with different degrees of pluripotency is the planar modulus $\bar{\mathbf{G}}^*_{\mathbf{2D}}$ measure in beads with time-independent (i.e. elastic) $\langle\langle\Delta r^2(\tau)\rangle\rangle_{2D}$ profiles [31]. Although we concur with the reported softness of hiPSCs, we found instead that beads with time-independent properties were particularly infrequent in hiPSCs when delivered by endocytosis. This would suggest that beads introduced cytoplasmically may show time-independent properties just as infrequently in cell types with lower degrees of stemness; if so, *in vitro* screening of multipotency based on rare instances of time-independent rheology may not only be inefficient, but also ill-posed to capture viscoelastic rheology inside differentiated cells.

Nevertheless, we do not rule out that using different bead delivery techniques to characterize a highly heterogeneous medium may confound subtle rheological differences between functionally distinct cytoplasmic compartments, e.g. microtubular networks or endoplasmic reticulum cisternae. To help address this possibility, further studies may be implemented to compare the spatial distribution of probes observed with each intracellular delivery technique, assess the scale-dependent behavior of intracellular rheology by using beads of different sizes, or characterize the mechanical footprint of nucleocytoskeletal connectivity disruption using exogenous treatments with predictable effects on structural components of cells.

**Cytokine signaling alters hASC cytoskeletal mechanics.** The effects of cytokine signaling on cytoskeletal remodeling have been widely investigated. We found that IL1α and TGFβ1, two cytokines with differential effects on cytoskeletal maintenance, elicited significant alterations on the mechanical profile of

hASCs (Figure 2a). Our results agree with previous reports of cytoskeletal remodeling effects induced by IL1α and TGFβ1 signaling [4,52]. Under this model, an enriched cell cortex comes at the expense of cytoskeletal fiber density in the cytoplasmic space, where F-actin from stress fibers around the nucleus is eroded; this process leads to perinuclear weakening against mechanical buckling and shearing. Our experimental observations match these predictions by showing radially enhanced anisotropy (higher **K**), preferential weakening of angular stiffness (loss in $\hat{\mathbf{G}}_{\mathbf{v}}$) and switch to a predominantly fluid-like behavior ($\log\check{\mathbf{N}} > 0$). In addition, our analysis also reveals significant effects on the structural organization of the pnCSK, leading to both a rounder nucleus (lower S) and sDL redistribution towards the major nuclear axis (lower **Ω**); such structural remodeling suggests that cells can ameliorate the effects from exogenous disruptors of cytoskeletal integrity by counteracting softening of the cytoskeleton with structural polarization of their pnCSK architecture (Figure 2b).

According to previous reports, the intracellular mechanical effects of IL1α and TGFβ1 in hASCs, although similar, arise from distinct intracellular kinetics and structural remodeling phenomena in the cytoskeleton. For example, TGFβ1 signaling can induce both myofibroblastic and chondrogenic specification, perhaps fine-tuning a choice between two lineages with different mechanical and morphological characteristics [52]. Our experimental findings are in line with those observations, with the added virtue that distinct treatment conditions can be readily discerned (Figure 2c). Given this backdrop, cytoskeletal mechanics after TGFβ1 induction are useful measures for hASC characterization because they scrutinize the onset of short-term cytoskeletal remodeling during designed *in vitro* TGFβ1 abundance.

**Constraints on cell morphology limit the mechanical adaptability of hASCs to external cues.** Disrupted cytoskeletal assembly results in magnified and compensatory structural remodeling of the pnCSK, particularly when an imposed cell morphology can constrain the shape of the nucleus [33]; our results using imposed cell shapes via μPP cultures agree with such evidence (Figures 3a and 3b); yet, morphological constraints also abrogate the ability to compile mechanical parameters into discriminant scores that can recognize the onset of cytoskeletal remodeling, such as CytoD induction (Figure 3c). Nonetheless, discriminant power is recovered under exogenous cytoskeletal induction on cells with naïve morphologies, such as those shown in Experiments I and III (Figures 2 and 4). Put together, our findings suggest that mechanical adaption in cells occurs by active modulation of anisotropic bias achieved through pnCSK remodeling, particularly when nuclear shape is morphologically restricted.

**Structural pnCSK mechanics are rapidly set and stabilized during active F-actin remodeling.** Our tests examined changes in cytoskeletal mechanics of hASCs dominated by short-term structural kinetics of F-actin polymerization. The observed IL1α-induced rheology was concordant with previously reported pnCSK fluidization through active cortical segregation of actin monomers [4]; in contrast, pnCSK thickening and anisotropic losses observed after Cytochalasin D treatments agree with a putative accumulation of G-actin monomers, due to F-actin microfilament capping and CD-actin dimerization [54].

One notable finding was that the structural properties of the pnCSK, although rapidly altered, did not evolve significantly for the next 2 hr after induction. In contrast, live imaging of tested cells (Figure 4a) confirmed a continued degradation of gross cytoskeletal features under both F-actin polymerization blockage (Cytochalasin D treatments) and inflammatory conditions (IL1α signaling). This dichotomy suggests our rheological characterization is specific to sDL, which might correspond to a highly stable cytoskeletal subdomain impervious to exogenously induced F-actin disassembly.

As mentioned earlier, observations from hASCs treated with CytoD led to notably distinct responses in stiffness, anisotropy and sDL distance from the nucleus for hASCs with or without patterned morphologies (Experiments I and II, respectively). In fact, the ability to discriminate between control and CytoD-treated cells was null in hASCs with directed morphology and yet highly significant in unpatterned hASCs. In our view, such discrepancies highlight negative effects of constrained cell morphology on the capacity of cells to adapt their mechanical profiles to resist exogenous stress (Figure 4c).

**Stress-strain relations couple pnCSK and nucleus as a single mechanical unit.** To translate the relevance of implementing our model in PTM data analysis from intracellular mechanics experiments, we now transform the elliptical map of pnCSK mechanics back into a rectangular coordinate system. In doing so, we can: a) illustrate how changing pnCSK mechanics can be teased out as portrayed by their effects on nuclear shape; and b) describe testing conditions for confirmatory experiments whose morphological and mechanical effects in cell mechanics can be weighed against the predicted effects from our pnCSK mechanics model. To do this, we compare the nuclear shape **S** to the relative normalized distance of sDL from the nucleus $\bar{r}=(x_B^2+y_B^2)^{1/2}/M$ with respect to the major nuclear axis and its position angle $\theta=\tan^{-1}(y_B/x_B)$. This allows to calculate relative distances $\Delta=\bar{r}-\bar{r}_{NE}$ between nucleus and sDL normalized to the major nuclear axis $M$.

To quantify coupling with the nucleocytoskeleton, a relation between the relocation of perinuclear subdiffusive sites and accompanying changes in nuclear shape can be estimated. Such relation can be fit

empirically through a variable power law such that $\mathit{\Delta} = 0.20\cdot\mathbf{S}^{0.17}\cdot[\bar{r}]^{ß}$, where $ß=ß(\bar{r})=2.9–4.3\cdot\ln(\bar{r})$ describes the ratio between rates of changing perinuclear gap and sDL position distances, respectively ($R^2$=0.84, $p$<0.0001). Furthermore, after introducing differentiation in time and true strain relations (e.g. $\varepsilon_{\mathit{\Delta}}\equiv d\mathit{\Delta}/\mathit{\Delta}$), we find that the instantaneous strain rate for the perinuclear gap distance depends on both the scale of sDL position and nuclear shape changes as $\varepsilon_{\mathit{\Delta}}=0.17\cdot\varepsilon_{\mathbf{S}}+[2.9–8.6\cdot\ln(\bar{r})]\cdot\varepsilon_{\bar{r}}$.

In principle, the empirical power-law relation between perinuclear gap, sDL position and nuclear shape can be used to posit, under a quantitative scheme, how the physical interactions between the nucleus and pnCSK would evolve under loading or mechanotransduction processes, thus offering a new predictive tool for hypothesis generation. For illustrative purposes, consider a putative pnCSK remodeling cycle A→B→C→D→A (Supporting Figure S2a) in a cell with both an initially elongated nucleus (Φ≈2, i.e. **S**≈6.5), and sDL position near the nucleus ($\log\bar{r}\approx–0.2$, i.e. $\bar{r}\approx0.6$) but away from the major axis (tanθ>1, i.e. θ>45°). First, during A→B, the elongated nuclear shape is conserved ($\Phi_A\approx\Phi_B$) while sDL move away from the nuclear center (↑$\bar{r}$) and towards the nuclear major axis (↓tanθ); this transition leads to a combined effect in which the perinuclear gap widens (↑$\mathit{\Delta}$) at a decelerating rate that, nonetheless, is always faster than the growing sDL distance from the nuclear center (↓$ß$ with $ß$>1 throughout). Next, for B→C with a constant position angle $\tan\theta_B\approx\tan\theta_C$, the nucleus rounds up (↓Φ) without increasing the strain ratio between the perinuclear gap and sDL position ($ß_B\approx ß_C$); this is possible if the pnCSK contracts preferentially along the major axis, thereby increasing the normalized sDL position (↑$\bar{r}$) while maintaining a similar proportion in the perinuclear gap ($\mathit{\Delta}_B\approx\mathit{\Delta}_C$). Then, during C→D, the rounded nucleus conserves its shape ($\Phi_C\approx\Phi_D$) as the pnCSK contracts in a manner opposite to the initial A→B (i.e. ↓$\bar{r}$, ↑tanθ, ↓$\mathit{\Delta}$, and ↑$ß$ with $ß$>1); and fourth, for the end-of-cycle transition D→A near the minor axis ($\tan\theta_D\approx\tan\theta_A$), the original pnCSK-nucleus configuration recovers by opposing the morphological changes introduced during the B→C stage (i.e. ↑Φ, ↓$\bar{r}$, $\mathit{\Delta}_D\approx\mathit{\Delta}_A$, $ß_D\approx ß_A$). Qualitatively, this putative cycle could be summarized into four hypothetical structural processes as follows: packed pnCSK unjamming and extension (A→B); nuclear rounding with major axis contraction (B→C); distended pnCSK relief and retraction (C→D); and nuclear flattening with major axis elongation (D→A).

**Cytoplasmic sDL lie along directions of principal shear stress through the center of the cell nucleus.** To communicate the structural implications of our pnCSK rheological model, it is helpful to interpret its predicted mechanical behavior at the linear viscoelastic limit (infinitesimal strains) also under a rectangular frame of reference; such an effort requires geometric elliptical-to-Cartesian transformation of the complex shear

modulus tensor $\tilde{\mathbf{G}}_{\mathbf{uv}}$*****, defined in the 2D case by two translational ($\mathbf{G_u}$*****, $\mathbf{G_v}$*****) and one rotational ($\mathbf{T_{uv}}$*****) components (see later in Supplementary Information for further details on mathematical derivations).

Assuming negligible rotational shear or curl in each local elliptical basis (i.e. $\mathbf{T_{uv}}^*=0$), the unique properties of our model reduce the complex shear modulus tensor $\tilde{\mathbf{G}}_{\mathbf{uv}}^*$ into two trace shear moduli ($\mathbf{G_u}^*$ and $\mathbf{G_v}^*$) with proportionally related amplitudes (anisotropic ratio $\mathbf{K}=\mathbf{G_u}^*/\mathbf{G_v}^*$) and constant phase (viscoelastic damping $\mathbf{G''/G'}=\tan[\alpha\cdot\pi/2]$), such that $\mathbf{G_u}^*=½\cdot(\mathbf{K}+1)\cdot\bar{\mathbf{G}}_{\mathbf{2D}}^*\cdot \boldsymbol{f}^{\check{\mathbf{n}}}\cdot e^{i\cdot\alpha\cdot\pi/2}$ and $\mathbf{G_v}^*=\mathbf{G_u}^*/\mathbf{K}$. From this starting point, we estimated the resultant phase between rectangular components (major axis $M \rightarrow x$-axis) for: a) principal pnCSK shear stress ratio $\sigma_m/\sigma_M$ under outwards equiaxial elliptical strain ($\varepsilon_u=\varepsilon_v>0$); and b) translational pnCSK shear strain ratio $\varepsilon_M/\varepsilon_m$ under outwards equiaxial Cartesian stress ($\sigma_m=\sigma_M>0$).

According to our empirical model of pnCSK mechanics, the minor-to-major nuclear axes stress ratio $\sigma_m/\sigma_M$ at a sDL in the pnCSK when subjected to elliptical equiaxial strain $\varepsilon_u=\varepsilon_v$ away from the nucleus is equal to the ratio between the minor and major nuclear axes tensorial components of $\tilde{\mathbf{G}}_{\mathbf{Mm}}^*$ and relates to the position angle θ of the sDL as $\sigma_m/\sigma_M \equiv G_m/G_M \geq \tan(\theta)$; in other words, the transmission line of the principal shear stress transects the nuclear center (Supporting Figure S2b). This collinearity between the pnCSK principal shear stress direction and the center of the nucleus suggests that sDLs are candidate stress-bearing sites in the cytoplasm, which resist and dampen mechanical forces aimed directly towards the nucleus.

Conversely, when the pnCSK at a sDL experiences rectangular equiaxial stress $\sigma_m=\sigma_M$ pulling outwards (i.e. equal forces in orthogonal directions parallel to either nuclear axis) the average resultant strain is collinear with the center of the nucleus if the sDL position angle lies between 30.4° and 56.6° from the major axis; outside this range, the resulting outwards strain favors deformations along the minor axis when θ<30.4° – i.e. cells with elongated nuclei tend to round up – or along the major axis when θ>56.6° – i.e. cells with rounded nuclei tend to flatten down (Supporting Figure S2c).

One noteworthy prediction from our model reveals that, if pnCSK mechanics exhibit orthotropic characteristics, then mechanical analyses using rectangular coordinates can conceal the distinctions between translation and rotational shear strains at sDL, whereas a nucleus-centered elliptical coordinate system readily distinguishes them because it explicitly accounts for cell morphology. This discrepancy is best illustrated by tensor transformation of shear modulus $\tilde{\mathbf{G}}_{\mathbf{uv}}^*\rightarrow\tilde{\mathbf{G}}_{\mathbf{Mm}}^*$ as follows: given an elliptical model of pnCSK mechanics with negligible elliptical strain curl ($\varepsilon_{uv}=0$) and anisotropic properties ($\mathbf{K}>1$), it can be shown by geometric transformation that the rectangular stress tensor $\tilde{\mathbf{G}}_{\mathbf{Mm}}^*$ contains a rotational shear modulus relative to

rectangular coordinates $\mathbf{T_{Mm}}$*=[$\bar{\mathbf{G}}_{\mathbf{2D}}$*·(1–$\mathbf{K}^2$)/(4·$\mathbf{K}$)]·sin{2·tan$^{-1}$[$\mathbf{\Omega}^2$/tan(θ)]}. This tensorial property is experimentally significant, since it predicts that any translational pnCSK strain under outwards equiaxial stress will evoke a clockwise rotational shear strain *with respect to a Cartesian frame of reference* – i.e. small deformations curling towards the major nuclear axis. Conversely, it is possible to measure a rotational shear in pnCSK when using Cartesian coordinates that, in fact, may equal no-curl conditions in elliptical strain.

**Geometric transformation of rheological metrics between elliptical and Cartesian coordinates.** Consider a two-dimensional elliptical coordinate system with unit basis $\{\boldsymbol{u},\boldsymbol{v}\}$ and interfocal length $\boldsymbol{2f}$. Next, superimpose a Cartesian coordinate system at the origin with unit basis $\{\boldsymbol{x},\boldsymbol{y}\}$ and $x$-axis lying on the interfocal trace of the elliptical coordinate system. The geometric transformation matrix $\mathbf{Q}$ that maps between $\{\boldsymbol{u},\boldsymbol{v}\}$ and $\{\boldsymbol{x},\boldsymbol{y}\}$ at position vectors $[x\ y]^{\mathrm{T}}$ and $[u\ v]^{\mathrm{T}}$ such that

$$\begin{bmatrix} x \\ y \end{bmatrix} = \mathbf{Q} \begin{bmatrix} u \\ v \end{bmatrix}$$

for $x = f\cosh(u)\cos(v)$ and $y = f\sinh(u)\sin(v)$ is equal to the Jacobian matrix

$$\mathbf{Q} = \begin{bmatrix} \frac{\partial x}{\partial u} & \frac{\partial x}{\partial v} \\ \frac{\partial y}{\partial u} & \frac{\partial y}{\partial v} \end{bmatrix} = \frac{1}{\sqrt{D}} \begin{bmatrix} \sinh(u)\cos(v) & -\cosh(u)\sin(v) \\ \cosh(u)\sin(v) & \sinh(u)\cos(v) \end{bmatrix}$$

where $D = \frac{1}{2}f^2[\cosh(2u) - \cos(2v)]$ is a curvilinear scale factor. Furthermore, an elliptical second-order tensor $\tilde{\mathbf{E}}$ maps to its Cartesian equivalent $\tilde{\mathbf{C}}$ by means of a change-of-basis operation that involves the geometric transformation matrix **Q:**

$$\tilde{\mathbf{C}} = \mathbf{Q}^{\mathbf{T}} \cdot \tilde{\mathbf{E}} \cdot \mathbf{Q}$$

Thus, the elliptical shear modulus tensor $\tilde{\mathbf{G}}_{\mathbf{uv}}$ defined in terms of translational shear moduli $\mathbf{G}_{\boldsymbol{u}}$ and $\mathbf{G}_{\boldsymbol{v}}$ in the $\boldsymbol{u}$ and $\boldsymbol{v}$ directions and rotational shear modulus $\mathbf{T}_{\boldsymbol{uv}}$ such that

$$\tilde{\mathbf{G}}_{\boldsymbol{uv}} = \begin{bmatrix} \mathbf{G}_{\boldsymbol{u}} & \mathbf{T}_{\boldsymbol{uv}} \\ \mathbf{T}_{\boldsymbol{uv}} & \mathbf{G}_{\boldsymbol{v}} \end{bmatrix}$$

maps into its Cartesian counterpart, shear modulus tensor $\tilde{\mathbf{G}}_{\boldsymbol{xy}} = \tilde{\mathbf{G}}_{\boldsymbol{Mm}}$ as

$$\tilde{\mathbf{G}}_{\boldsymbol{xy}} = \tilde{\mathbf{G}}_{\boldsymbol{Mm}} = \mathbf{Q}^{\mathbf{T}} \cdot \tilde{\mathbf{G}}_{\boldsymbol{uv}} \cdot \mathbf{Q} = \begin{bmatrix} \mathbf{G}_{\boldsymbol{M}} & \mathbf{T}_{\boldsymbol{Mm}} \\ \mathbf{T}_{\boldsymbol{Mm}} & \mathbf{G}_{\boldsymbol{m}} \end{bmatrix}$$

where we have renamed the basis $\{\boldsymbol{x}, \boldsymbol{y}\}$ as $\{\boldsymbol{M}, \boldsymbol{m}\}$ to correspond with the directions of the major and minor elliptical axes $\boldsymbol{M}$ and $\boldsymbol{m}$, respectively, as determined by the interfocal trace of the elliptical coordinate system.

**Mechanical observations predicted by infinitesimal stress-strain analysis of elliptical model for pnCSK mechanics as mapped to a Cartesian frame of reference.** *Equiaxial elliptical strain.* Assume an infinitesimal stress-strain relation such that

$$\boldsymbol{\sigma}_{\mathbf{E}} = \widetilde{\mathbf{G}}_{\boldsymbol{uv}} \cdot \boldsymbol{\varepsilon}_{\mathbf{E}}$$

$$\boldsymbol{\sigma}_{\mathbf{E}} = \begin{bmatrix} \sigma_u & \sigma_{uv} \\ \sigma_{vu} & \sigma_v \end{bmatrix} \cdot \begin{bmatrix} u \\ v \end{bmatrix}$$

$$\boldsymbol{\varepsilon}_{\mathbf{E}} = \begin{bmatrix} \varepsilon_u & \varepsilon_{uv} \\ \varepsilon_{vu} & \varepsilon_v \end{bmatrix} \cdot \begin{bmatrix} u \\ v \end{bmatrix}$$

for stress and strain vectors $\boldsymbol{\sigma}_{\mathbf{E}}$ and $\boldsymbol{\varepsilon}_{\mathbf{E}}$, respectively. Thus, given a negligible rotational elliptical shear strain ($\varepsilon_{uv} = 0$), the elliptical stress vector equals

$$\boldsymbol{\sigma}_{\mathbf{E}} = \begin{bmatrix} \mathbf{G}_{\boldsymbol{u}}\varepsilon_u & 0 \\ 0 & \mathbf{G}_v\varepsilon_v \end{bmatrix} \cdot \begin{bmatrix} u \\ v \end{bmatrix}$$

Given a principal stress condition with equiaxial elliptical strain $\varepsilon_u = \varepsilon_v$, the scalar elliptical stress ratio $\frac{\sigma_u}{\sigma_v}$ at the Cartesian position vector $[x\ y]^{\mathrm{T}}$ can be reformulated as $\frac{\sigma_M}{\sigma_m}$ by geometric transformation of the elliptical stress vector $\boldsymbol{\sigma}_{\mathbf{E}}$; therefore,

$$\begin{bmatrix} \sigma_M \\ \sigma_m \end{bmatrix} = \mathbf{Q} \begin{bmatrix} \sigma_u \\ \sigma_v \end{bmatrix} = \mathbf{Q} \begin{bmatrix} \mathbf{G}_{\boldsymbol{u}}\varepsilon_u \\ \mathbf{G}_v\varepsilon_v \end{bmatrix}$$

which reduces to

$$\tan\gamma_\sigma = \frac{\sigma_m}{\sigma_M} = \frac{\mathbf{K} \cdot \boldsymbol{\Omega}^2 + \tan\theta}{\mathbf{K} \cdot \tan\theta - \boldsymbol{\Omega}^2}$$

where $\mathbf{K} = \mathbf{G}_{\boldsymbol{u}}/\mathbf{G}_v$ and $\boldsymbol{\Omega} = \tan(v)$ are the anisotropic and hyperbolic indices from our nondimensional analysis shown previously, $\tan\theta = \frac{\mathrm{y}}{\mathrm{x}}$ is the rectangular position vector phase, and $\tan\gamma_\sigma = \frac{\sigma_m}{\sigma_M}$ is the translational shear stress phase.

*Equiaxial Cartesian stress.* Consider an equiaxial Cartesian stress condition with no rotational stress component and a negligible rotational shear modulus such that $\sigma_{Mm} = 0$ and $\mathbf{T}_{uv} \rightarrow 0$ (i.e. $\mathbf{T}_{Mm} \ll \mathbf{G}_{M}$ and $\mathbf{T}_{Mm} \ll \mathbf{G}_{m}$). Thus, both the translational strain ratio $\frac{\varepsilon_M}{\varepsilon_m}$ and the rotational strain or curl $\varepsilon_{Mm}$ as observed in Cartesian coordinates can be solved from the infinitesimal stress-strain relation in terms of elliptical metrics:

$$\boldsymbol{\sigma}_{\mathbf{C}} = \widetilde{\mathbf{G}}_{Mm} \cdot \boldsymbol{\varepsilon}_{\mathbf{C}} = \left[\mathbf{Q}^{\mathbf{T}} \cdot \widetilde{\mathbf{G}}_{uv} \cdot \mathbf{Q}\right] \cdot \begin{bmatrix} \varepsilon_M & \varepsilon_{Mm} \\ \varepsilon_{Mm} & \varepsilon_m \end{bmatrix} \cdot \begin{bmatrix} u \\ v \end{bmatrix}.$$

Hence, the translational strain ratio in Cartesian coordinates reduces to

$$\tan \gamma_{\varepsilon} = \frac{\varepsilon_m}{\varepsilon_M} = \frac{\mathbf{K} \cdot \left(\frac{\tan \theta}{\boldsymbol{\Omega}}\right)^2 + \boldsymbol{\Omega}^2}{\mathbf{K} \cdot \boldsymbol{\Omega}^2 + \left(\frac{\tan \theta}{\boldsymbol{\Omega}}\right)^2}$$

where $\tan \gamma_{\varepsilon} = \frac{\varepsilon_m}{\varepsilon_M}$ is the translational shear strain phase. Furthermore, for a power-law rheological model with frequency-invariant viscoelastic damping and constant anisotropic index $\mathbf{K}$, the nonzero Cartesian rotational shear modulus $\mathbf{T}_{Mm}$ at $[x\ y]^{\mathrm{T}}$, equals

$$\mathbf{T}_{Mm} = \mathbf{G}_{\mathbf{2D}} \cdot \left(\frac{1 - \mathbf{K}^2}{4\mathbf{K}}\right) \cdot \sin\left[2 \cdot \tan^{-1}\left(\frac{\boldsymbol{\Omega}^2}{\tan \theta}\right)\right] \leq 0$$

i.e. towards the major axis of the elliptical coordinate system, where $\mathbf{G}_{\mathbf{2D}}$ is the planar shear modulus as estimated via the Stokes Einstein relation such that

$$\langle\langle r_{\mathbf{2D}}^2 \rangle\rangle = \langle\langle r_u^2 \rangle\rangle + \langle\langle r_v^2 \rangle\rangle$$

which yields

$$\mathbf{G}_{\mathbf{2D}} = 2\left(\frac{\mathbf{G}_{u} \cdot \mathbf{G}_{v}}{\mathbf{G}_{u} + \mathbf{G}_{v}}\right).$$

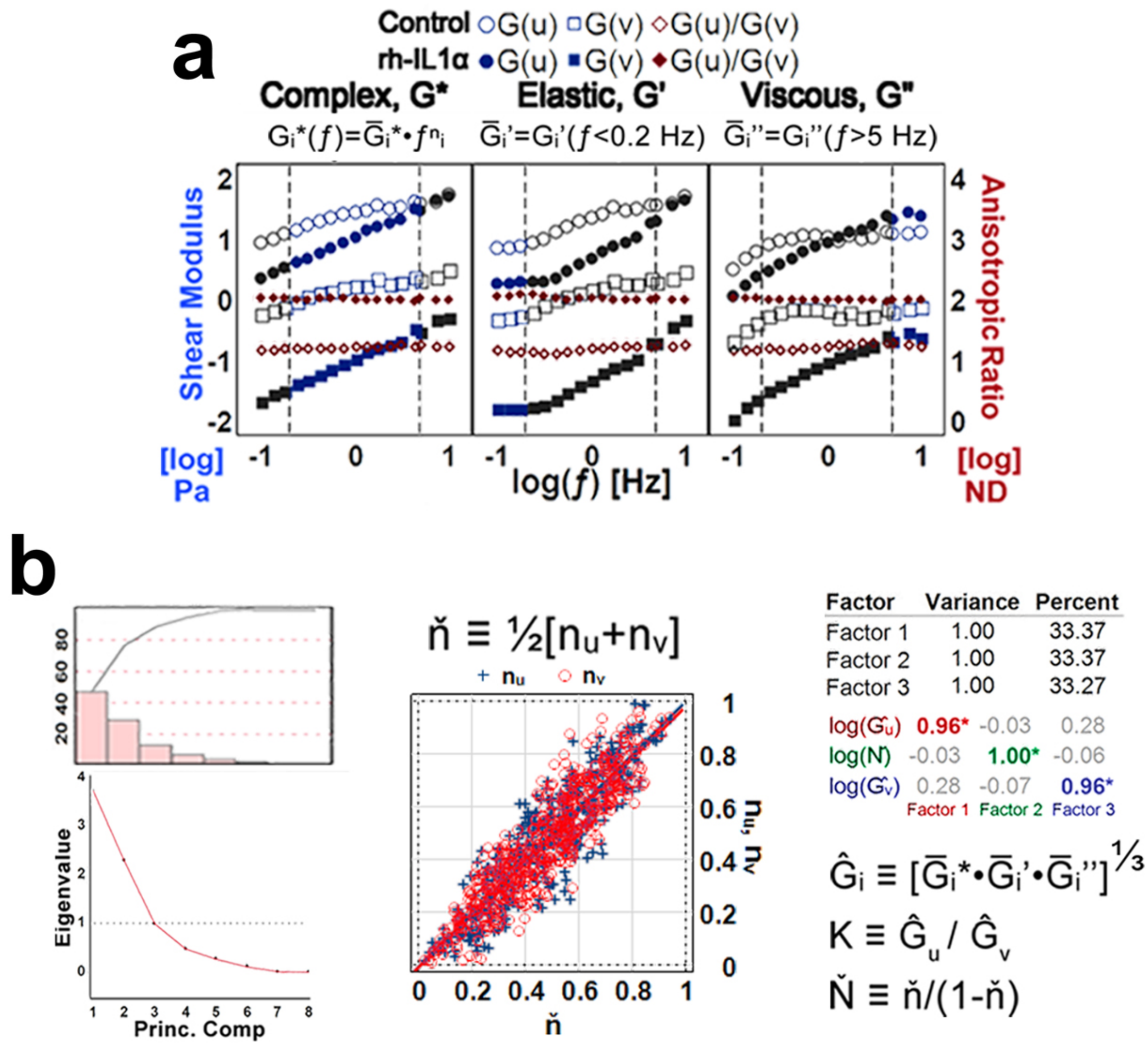


**Supporting Figure S1. Rheological characterization of the perinuclear cytoskeleton (pnCSK) in elliptical coordinates.** a) Complex shear moduli $\mathbf{G_i^*}$ exhibit a consistent power-law rheology (flow consistency and flow behaviour indices $\mathbf{\bar{G}_i^*}$ and $\mathbf{n_i}$ for $\mathbf{i}=\{\mathbf{u},\mathbf{v}\}$) in the frequency domain 0.2 Hz<$\boldsymbol{f}_P$<5 Hz, which is bounded by elastic and viscous shear moduli plateaus $\{\mathbf{\bar{G}_i'},\mathbf{\bar{G}_i''}\}$; such rheological behavior is conserved under various exogenous conditions, including rhIL-1α supplementation at 10 ng/ml. b) PCA of rheological parameters in elliptical coordinates (Kaiser criterion) reduces cytoskeletal properties into 3 orthogonal factors with equal variance: determinant shear moduli $\mathbf{\hat{G}_u}$ and $\mathbf{\hat{G}_v}$ with anisotropic ratio $\mathbf{K}\equiv\mathbf{\hat{G}_u}/\mathbf{\hat{G}_v}$; and fluidity $\mathbf{\check{N}}\equiv\mathbf{\check{n}}/(1-\mathbf{\check{n}})$, equal to the logistic ratio of the mean flow behavior index, or power-law exponent, $\mathbf{\check{n}}\equiv\frac{1}{2}(\mathbf{n_u}+\mathbf{n_v})$ calculated from flow behavior indices $\mathbf{n_u}$ and $\mathbf{n_v}$ that exhibit one-to-one correspondence and statistical equivalence.

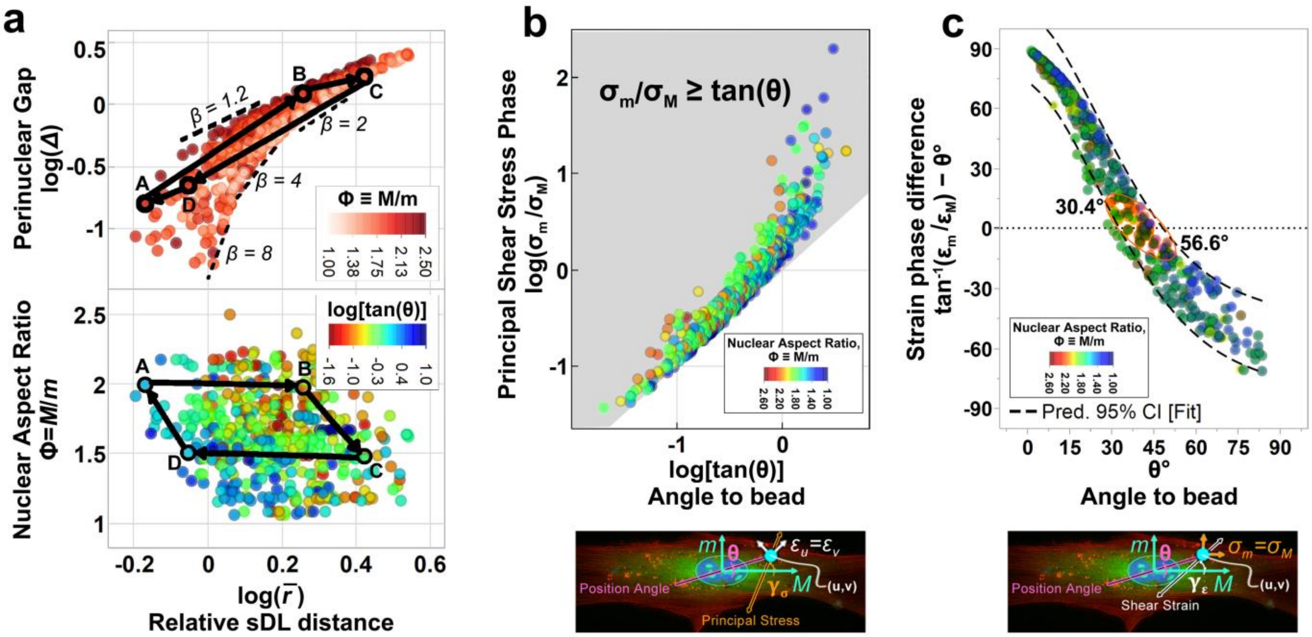


**Supporting Figure S2. Principal shear modulus and mechanical states in the stem cell pnCSK.** a) Hypothetical strain 4-step cycle of the nucleus-pnCSK complex as a function of nuclear aspect ratio $\Phi = M/m$, Cartesian angle to bead tan(θ), relative sDL position $\bar{r} = r/M$ (nuclear perimeter at $r_{NE}$), relative perinuclear gap $\Delta = (r - r_{NE})/M = \bar{r} - \bar{r}_{NE}$ and relative pnCSK-to-nuclear strain ratio *ß*. b) The direction angle $\gamma_\sigma = \tan^{-1}(\sigma_m/\sigma_M)$ of the principal shear stress under equiaxial elliptical strain $\varepsilon_u = \varepsilon_v > 0$ for the pnCSK at sDL, with respect to the major and minor nuclear axes *M* and *m* is equal or greater than the phase tan(θ) for the sDL position, as depicted in accompanying cell diagram. c) Equiaxial rectangular stress $\sigma_m = \sigma_M > 0$ induces local strains at sDL with translational shear strain direction angle $\gamma_\varepsilon = \tan^{-1}(\varepsilon_m/\varepsilon_M) = \theta$ for 30.4°<θ<56.6° (prediction 95% confidence interval to $\gamma_\varepsilon - \theta = 0$ is highlighted in orange for clarity); otherwise, local strain effects exhibit either $\gamma_\varepsilon > \theta$ when θ<30.4° or $\gamma_\varepsilon < \theta$ when θ>56.6°.

## Supporting Table S1. Implemented testing configurations for particle-tracking microrheology (PTM).

| **Site** | CISMM (UNC at Chapel Hill) | | | | ORL (Duke University) | | | |
|---|---|---|---|---|---|---|---|---|
| **Configuration** | PTM+F | | | | PTM+C | | | |
| **Microscope** | Nikon TE 2000 Inverted Microscope | | | | Zeiss LSM 510 Laser Scanning Inverted Microscope | | | |
| **Objective** | 100X/1.3NA oil, Nikon Plan Fluor-DIC-H | | | | EC Plan-Neofluar 40X/0.75 Ph2 | | | |
| **Port/Modular Magnification** | 10X port | | | | 10X port<br>+ 2X DL tube | | 10X port<br>+ 2.5X upright telescope<br>+ 4.1X digital zoom | |
| **Experiment** | I: Anabolic v. Catabolic Cytokine Induction<br>(TGFβ1, IL1α) | | II: Directed v. Naïve Cell Morphology<br>(±15 μm×70 μm FN-coated μPP; ±CytoD) | | III: Inflammation v. Actin Depolymerization<br>(Time-Lapse: IL1α, CytoD) | | IV: Human Stem Cell Signatures<br>(hiPSC, hMSC, hASC) | |
| **Graphics Data** | Image | Video | Image | Video | Image | Video | Image | Video |
| **DAQ Scan Settings**<br>**[Time/Rate]** | Progressive CCD; brightfield, fluorescence:<br>DAPI<br>FITC-eGFP<br>Texas Red<br>[single-frame:<br>50ms, 2X gain] | Progressive CCD; brightfield<br>[60 s / 120 fps] | Progressive CCD; brightfield<br>[single frame:<br>1 s / 1 fps] | Progressive CCD; brightfield<br>[60 s / 120 fps] | Multi-beam line scanning; Ex(λ)/Em(filter):<br>488/BP505-550<br>543/BP560-615<br>633/LP650 | Progressive CCD; brightfield<br>[30 s / 100 fps] | Multi-beam line scanning; Ex(λ)/Em(filter):<br>488/BP505-550<br>543/BP560-615<br>633/LP650 | Progressive CCD; brightfield<br>[15 s / 30 fps] |
| **DAQ System** | Roper<br>Cascade II:512<br>(16-bit, mono) | JAI/Pulnix<br>CM-030GE-RH<br>(8-bit, mono) | | | Zeiss<br>Laser Confocal/DIC | Prosilica<br>GX1050<br>(8-bit, mono) | Zeiss<br>Laser Confocal/DIC | Prosilica<br>GX1050<br>(8-bit, mono) |
| **Digital Resolution** | 0.16 μm/px | 0.090 μm/px | | | 0.45 μm/px | 0.073 μm/px | 0.055 μm/px | |
| **Frame: L×W, px** | 512 × 512 | 648 × 484 | | | 512 × 512 | 1024 × 1024 | | |

## Supporting Table S2. Qualitative effects of exogenous treatments on pnCSK mechanics in hASCs.

| **Exp ID** | **Cell Shape** | **Treatment** | **Effect Size v. Untreated (hASCs)** | | | | | | | |
|---|---|---|---|---|---|---|---|---|---|---|
| | | | **Shear Modulus (Pa)** | | | **Rheological Factors (ND)** | | **Bead Mapping Factors (ND)** | | |
| | | | $\bar{G}_{2D}^*$ | $\hat{G}_u$ | $\hat{G}_v$ | **K** | **Ň** | **S** | **Ɽ ≡ Θ−1** | **Ω** |
| I | Naïve | IL-1α, 10 ng/ml | ⇩ | ⇩ | ⇩ | ⇧ | ⇧ | ⇩ | ⇔ | ⇩ |
| | | TGFß1, 10 ng/ml | ⇩ | ⇩ | ⇩ | ⇔ | ⇧ | ⇔ | ⇔ | ⇩ |
| II | μPP | CytoD, 0.5 μM | ⇧ | ⇔ | ⇧ | ⇩ | ⇔ | ⇔ | ⇩ | ⇧ |
| III | Naïve | IL-1α, 10 ng/ml | ⇩ | ⇩ | ⇩ | ⇧ | ⇧ | ⇩ | ⇩ | ⇩ |
| | | CytoD, 0.5 μM | ⇔ | ⇔ | ⇔ | ⇩ | ⇧ | ⇩ | ⇩ | ⇧ |
